# Dissection of local haplotype diversity at soybean rust loci reveals resistance-associated and context-dependent variation patterns in diverse germplasm

**DOI:** 10.1007/s00122-026-05209-6

**Published:** 2026-03-25

**Authors:** Shameela Mohamedikbal, Hawlader A. Al-Mamun, Jacob I. Marsh, Shriprabha R. Upadhyaya, Jacqueline Batley, David Edwards

**Affiliations:** 1 Centre for Applied Bioinformatics, University of Western Australia, Perth, WA 6009 Australia; 2 School of Biological Sciences, University of Western Australia, Perth, WA 6009 Australia; 3 InterGrain Pty Ltd, Perth, WA 6163 Australia; 4 Department of Biology, University of North Carolina, Chapel Hill, NC 27599 USA

## Abstract

**Key message:**

Soybean rust-associated haplotypes around significant SNPs show variable effects across diverse accessions, while distinct soybean mosaic virus-resistant haplotypes were identified at the *Rsv1* locus on chromosome 13.

**Abstract:**

Soybean rust (SBR), caused by the fungal pathogen *Phakopsora pachyrhizi,* significantly affects soybean yield and quality globally. Here, we present an association and haplotype analysis of 2,815 phenotypically diverse soybean accessions to identify durable sources of genetic variation associated with SBR. We characterised allelic diversity and local haplotype effects at two important SBR loci on chromosomes 13 and 18 (*Rpp1*). At these genomic regions, marker groups containing tightly linked single-nucleotide polymorphisms (SNPs) associated with reduced disease severity were identified. In accessions showing reddish-brown lesion, marker groups associated with increased disease severity include gene variants in *Glyma.18G280400* and *Glyma.18G280300,* indicating potential for resistance improvement. Within the delimited genomic region on chromosome 13, which is reported to be associated with multiple soybean diseases, we also mapped resistance-specific haplotypes associated with soybean mosaic virus, including in the *Rsv1* carrier PI 96983, identifying candidate genes that may contribute to resistance. Cross-population haplotype transfer analysis between reddish-brown and tan lesion accessions for SBR around all significant loci showed context dependence of variation patterns, with stable loci on chromosome 07, having gene variants for *Glyma.07G261000*. These findings provide insights into the genetic architecture associated with soybean disease resistance and contribute to haplotype-based resistance breeding efforts; however, future functional validation of identified candidate causal alleles and genes is required. Our results also demonstrate the potential of local haplotyping with newer phenotypic data for SBR to identify linked causal alleles and individuals containing beneficial alleles, for breeding applications.

**Supplementary Information:**

The online version contains supplementary material available at 10.1007/s00122-026-05209-6.

## Introduction

Plant pests and pathogens significantly affect soybean (*Glycine max* (L.) Merr.) yield and seed quality globally. Soybean rust (SBR), caused by the biotrophic fungal pathogen *Phakopsora pachyrhizi* Sydow & P. Sydow*,* is a destructive foliar disease, especially under environmental conditions favourable to the pathogen such as high relative humidity and moderate temperatures (Goellner et al. 2010; Hossain et al. 2023). In uncontrolled outbreaks, SBR can cause yield losses of 50–90%, as reported in Brazil, Indonesia, and parts of Africa (Godoy et al. 2016; Sumartini and Sari 2022; Favoretto et al. 2025). With climate change expected to increase pathogen virulence and spread (Singh et al. 2023), milder winters and changes in relative humidity may enable *P. pachyrhizi* to expand into new geographical areas where it is currently not a significant concern (Chicowski et al. 2024)

Resistance to *P. pachyrhizi* is characterised either by an immune reaction with no visible lesions or reddish-brown (RB) lesions (Bromfield 1984). RB resistance, which generally shows sparse uredinium development compared to susceptible tan (TAN) reaction, can display a spectrum of reaction types, varying in sporulation levels and lesion colour (Bromfield 1984; Bonde et al. 2006). Similarly, TAN coloured lesions also show gradations based on sporulation (Bromfield 1984; Miles et al. 2011). However, it has been noted in previous studies that lesion colour (RB or TAN) by itself may not be an objective measure of resistance, as the number of uredinia per lesion and sporulation levels are not necessarily correlated with the lesion colour (Yamanaka et al. 2010, 2015b). Partial resistance (slow rusting or rate-reducing resistance) can occur within TAN phenotypes, resulting in reduced fungal colonisation or sporulation compared to fully susceptible types (Miles et al. 2011). The variation within RB and TAN sporulation levels necessitates genomic approaches that preserve the full phenotypic gradient rather than reducing resistance to a binary classification, to identify resistance-associated genetic variation patterns. *P. pachyrhizi* is highly adaptive; selective sweeps and genetic shifts within pathogen populations can lead to the emergence of new strains that overcome deployed monogenic *Rpp*-mediated resistance (Akamatsu et al. 2013; Paul et al. 2013; McDonald and Stukenbrock 2016; Gupta et al. 2023). Thus, identifying more durable sources of genetic variation is important to inform resistance breeding strategies.

Eight major *Rpp* (resistance to *P. pachyrhizi*) genes and numerous quantitative trait loci (QTL) associated with SBR have been characterised on various chromosomes using linkage mapping and genome-wide association studies (GWAS) (Hossain et al. 2015; Yamanaka et al. 2015a; Harris et al. 2015; Chang et al. 2016; Vuong et al. 2016; Walker et al. 2022; Lin et al. 2022; Xiong et al. 2023). Conventional single-locus GWAS models typically test the correlation of each single-nucleotide polymorphism (SNP) with the phenotype independently. However, self-pollinating species including soybean exhibit extensive linkage disequilibrium (LD) primarily because of reduced recombination under selfing, with domestication bottlenecks and intense selection for desirable traits further increasing LD (Hyten et al. 2007a; Li et al. 2013). Because GWAS resolution depends on the extent of historical recombination among individuals in an association panel, high LD can lead to noncausal SNPs appearing associated with a trait and the combined phenotypic effects of linked variants may remain underestimated or unresolved (Greenbaum and Deng 2017; Schaid et al. 2018; Tam et al. 2019; Michno et al. 2020). Since SNPs in high LD act as single units of variation (haplotypes) and can contribute to divergent phenotypes under selection, capturing their cumulative effects and potential epistatic interactions enables fine-scale refinement of trait-associated loci (Lewontin and Kojima 1960; Marsh et al. 2022b).

Local haplotyping, as a fine-mapping approach to GWAS, analyses groups of tightly linked, co-inherited SNPs within focused genomic regions to identify biologically meaningful patterns of functional variation around trait-associated loci (Qian et al. 2017; Belzile et al. 2020; Marsh et al. 2023). Haplotypes can be defined by quantifying pairwise LD between SNPs, typically using the squared correlation coefficient (*r*^2^) (Hill and Robertson 1968; Devlin and Risch 1995; Pritchard and Przeworski 2001). LD-based gene- and site-centric haplotyping has enabled finer resolution of loci influencing agronomic traits including seed weight, flowering time, oil, protein, branch number, and 100-seed weight in soybean (Tardivel et al. 2019; Torkamaneh et al. 2021; Marsh et al. 2022a; Bhat et al. 2022, 2025; Yu et al. 2024; Mohamedikbal et al. 2024; Kim et al. 2025). Identifying favourable haplotypes associated with a trait, moving beyond the individual contribution of a specific SNP, facilitates high-density molecular marker development which can enhance the efficiency of haplotype-based breeding strategies (Bhat et al. 2021; Sivabharathi et al. 2024). Additionally, significant associations between candidate haplotypes and phenotypes allow screening of candidate genes for coding or regulatory variants, further linking marker–trait associations with functional validation (Yu et al. 2025).

Characterisation of genomic regions associated with SBR has undergone several refinements. For instance, the *Rpp1* gene was first identified as a single dominant gene (McLean and Byth 1980; Hartwig and Bromfield 1983) and later mapped to soybean linkage group G (Hyten et al. 2007b) on chromosome 18. Subsequent studies detected the *Rpp1* locus in diverse accessions including those originating from Uganda, Malaysia, and India (Aoyagi et al. 2020; Paul et al. 2020; Ratnaparkhe et al. 2020). Previous studies have characterised allelic diversity at the *Rpp1* locus using SNP-based analyses or phylogenetic clustering, successfully identifying diagnostic allelic combinations that distinguish known resistant sources from susceptible checks in pre-selected germplasm and biparental mapping populations (Barros et al. 2023; Aoyagi et al. 2024). As Yamanaka et al. (2010) note, identifying such DNA markers or allelic patterns based on visual classification of RB/TAN may be useful to select lines from a population where major resistance genes segregate. A comprehensive mapping of haplotype diversity using LD-based approaches at the *Rpp1* locus in a large, unselected, phenotypically diverse germplasm enables characterisation of the full spectrum of genetic variation associated with different levels of RB and TAN reactions and clarify the linkage architecture underlying SBR resistance. Such LD-based approaches also enable determining whether haplotypes identified around significant SNPs in one phenotypic class (RB) transfer their beneficial effects when present in individuals showing TAN reactions within a germplasm panel, since reproducibility depends on the LD structure of the SNP with the causal allele.

A region on chromosome 13 (qSBR_Gm13) between 97.97 and 111.88 cM has been reported to be associated with SBR with a confidence interval of 11 cM (Harris et al. 2015). Notably, chromosome 13 is widely studied at the molecular level for understanding R-gene evolution, with the 28–32 Mb genomic region found to be highly enriched in resistance genes belonging to the nucleotide-binding-site leucine-rich repeat (NBS-LRR) family (Yu et al. 1996; Innes et al. 2008; Ashfield et al. 2012; Clevinger et al. 2024). This region is associated with resistance to multiple pathogens including bacteria, nematodes, and viruses such as the *Rsv1* locus for soybean mosaic virus (SMV) (Tamulonis et al. 1997; Ashfield et al. 1998; Gore et al. 2002; Hayes et al. 2004; Wu et al. 2019; Lin et al. 2022; Clevinger et al. 2024). Given the high density of R-genes in this region and potential haplotype diversity, LD-based haplotype analysis could reveal resistance-associated allelic variants that are shared across multiple pathogens such as SBR and SMV or alternatively identify pathogen-specific variants. Genomic regions with multiple defence genes and colocalised QTLs for different diseases may confer broad-spectrum protection and are valuable for addressing multiple breeding objectives simultaneously (Wiesner‑Hanks and Nelson 2016).

Here, we use an LD-based local haplotyping approach with high-density imputed SNP data to re-analyse publicly available genotypic and phenotypic datasets, to examine allelic diversity and linkage structures around previously identified resistance loci associated with SBR. Rather than assigning binary phenotypes (RB or TAN) a priori, we use rust severity scores (Miles et al. 2006) derived from lesion density for haplotype-phenotype association analysis. Our objectives were to: (i) characterise linkage structures and capture local haplotype diversity around GWAS-associated SBR loci on chromosomes 13 and 18 in a large, phenotypically diverse soybean germplasm panel; (ii) evaluate whether haplotypes associated with reduced disease severity in rust-resistant RB accessions exhibit similar phenotypic effects when present in susceptible TAN individuals; and (iii) identify and compare haplotypes associated with SBR and SMV around shared loci on chromosome 13 to reveal shared or pathogen-specific variation patterns.

Our results demonstrates that LD-based local haplotyping around important resistance loci using the full phenotypic gradient enables fine-scale dissection of allelic diversity underlying SBR resistance, identifying candidate linked causal alleles and individuals with beneficial alleles, for breeding applications and can be applied to newer SBR phenotyping data.

## Materials and methods

Throughout the manuscript, marker groups (MGs) refer to distinct groups of co-inherited SNPs that are in high LD with each other and can therefore be represented by a single data point. Haplotypes are defined as unique allelic combinations of MGs shared between individuals in the population studied.

## Plant material and phenotyping

A two-tiered greenhouse inoculation programme was previously conducted to evaluate resistance to SBR in the soybean accessions from the United States Department of Agriculture (USDA) germplasm collection using a mixture of four *P. pachyrhizi* isolates, described in detail by Miles et al. (2006). Disease severity was recorded as lesion density on a 1–5 scale, with lower scores (1) indicating no visible lesion development and higher scores (5) indicating prolific lesion development over most of the leaf area (Miles et al. 2006). Lesion colour was also recorded as RB (resistant), TAN (susceptible), or mixed reaction. Mean disease severity per genotype from the secondary evaluation with three replicates was used in the present study, as compiled in the USDA Germplasm Resources Information Network (USDA-GRIN) database, with 2,815 diverse soybean accessions (101 RB and 2,714 TAN reaction types). Mean severity score from Miles et al. (2006) evaluation has been used previously for association analyses and genomic prediction (Chang et al. 2016; Xiong et al. 2023). For GWAS and haplotype analysis, mean rust severity scores were transformed to a unified 1–9 scale, where 1 indicates fully resistant and 9 indicates fully susceptible reaction. A list of accessions, their raw disease severity ratings, and lesion colour used in this study is available in Table S1.

## Genotyping and quality control

Genotype data for the 2,815 accessions were obtained from the SoySNP50K iSelect BeadChip genotyping data (Song et al. 2013, 2015) aligned to the Williams 82 (Wm82.a2.v1) reference genome (Schmutz et al. 2010), with 42,080 SNPs. Biallelic SNPs with a minor allele frequency (MAF) > 0.05 with 35,191 SNPs were included for subsequent analyses.

## Imputation of missing SNPs in the SoySNP50K using a reference panel

Imputation increases SNP density in a study panel for association testing by leveraging shared haplotype information from a reference panel without requiring additional genotyping (Li et al. 2009). This approach has been widely used in genomic studies across species, including soybean, to improve the power of genetic analyses and to narrow down trait-associated loci (Chen et al. 2022; Potapova et al. 2025). In this study, the SoySNP50K dataset was phased and imputed using Beagle 5.4 (Browning et al. 2018, 2021) and a reference panel with 399 resequenced diverse soybean accessions (Valliyodan et al. 2021). Over 95% of the resequenced accessions showed high genetic similarity to their SoySNP50K counterparts (Valliyodan et al. 2021). This reference panel contained 7.8 million SNPs aligned to the Wm82.a2.v1 reference genome. To ensure reliable imputed variants, SNPs with a dosage R-squared (DR^2^ ≥ 0.8), which estimates the squared correlation between the imputed and true allele dosage, and MAF > 0.03 were retained, resulting in 1,321,040 SNPs for downstream analyses.

## Genome-wide association study for soybean rust resistance

Principal component analysis was performed using PLINK v1.9 (Purcell et al. 2007) to assess population structure. GWAS was conducted on the SoySNP50K dataset using Mixed Linear Model (MLM) (Yu et al. 2006) and Fixed and random model Circulating Probability Unification (FarmCPU) model (Liu et al. 2016) implemented in rMVP v1.1.1 (Yin et al. 2021) in R Statistical Software v4.3.3 (R Core Team 2024). Population structure was controlled using principal components in the model (nPC.MLM = 5, nPC.FarmCPU = 4). GWAS was also conducted on the imputed dataset using the FarmCPU model to uncover additional loci. The Bonferroni-corrected threshold for determining the statistical significance of the markers was set as 0.05 divided by the number of SNP markers, resulting in significance thresholds of 1.42 × 10^−6^ for SoySNP50K and 3.78 × 10^−8^ for the imputed dataset.

## Linkage disequilibrium analysis around trait-associated genomic regions

Linkage disequilibrium analysis was conducted using the imputed dataset to examine the local LD structure in regions of interest surrounding the GWAS-SNPs on chromosomes 13 and 18, to delimit genomic regions for local haplotyping. Pairwise LD statistics (squared correlation coefficient-r^2^) were calculated using PLINK v1.9 (Purcell et al. 2007) with -–r2 square parameter, generating an LD matrix for all SNPs within each region. LD heatmaps were generated using LDBlockShow v1.40 (Dong et al. 2021), with block boundaries defined using -BlockType 2. Both r^2^ and D′ patterns were visualised with -Selevar 3.

## Local haplotyping around the soybean rust-associated regions on chromosomes 13 and 18

Local haplotype analysis was conducted using an LD-based R package, crosshap v1.4.0 (Marsh et al. 2023), to identify haplotypes and map the local linkage landscape in genomic regions associated with SBR around the significant SNP on chromosomes 13 and 18. crosshap generates local haplotypes by clustering SNPs in strong pairwise LD (r^2^) into marker groups (MGs), which are typically co-inherited as a single unit within the population (Marsh et al. 2023). Individuals are then assigned to haplotype groups based on shared allelic combinations of these MGs. The haplotype window was delimited based on the local LD structure, as determined from PLINK and LDBlockShow outputs, and the proximity of the *Rpp1* locus on chromosome 18, and a QTL identified on chromosome 13. The lesion colour (RB or TAN) was used as metadata for the haplotyping analysis. crosshap was run with the following parameters: epsilon = 0.2, MGmin = 8 for the analysis on chromosome 18, and epsilon = 1.0, MGmin = 40 for chromosome 13. MGmin defines the minimum number of linked SNPs required to form an MG. Epsilon controls the clustering resolution. At lower epsilon values, only tightly linked SNPs are included in an MG. Haplotype analyses were conducted using the imputed dataset to ensure high SNP density to form haplotypes in the genomic regions studied.

## Cross-population marker group transfer analysis from RB to TAN accessions around soybean rust-associated loci

To investigate the transferability and phenotypic effects of resistance-associated haplotypes derived from RB on TAN populations, we used custom-modified scripts within the crosshap pipeline. These scripts bypass the original unsupervised LD clustering step of crosshap and instead fix the RB-derived MGs as input. Using these MGs, haplotypes were reconstructed in the TAN populations within the genomic regions surrounding all the significant SNPs obtained using GWAS with SoySNP50K. This enabled a direct comparison of haplotype composition and phenotypic effects between RB and TAN populations. Phenotypic differences in trait means of individuals having the alternate alleles for the MGs vs. those with reference alleles (phenodiff) and alternate allele frequency were used to evaluate the effects of RB-derived MGs within the TAN population.

## Local haplotyping in the focused genomic region on chromosome 13 for soybean mosaic virus (SMV-G1) resistance

Loci associated with SMV strain G1 (SMV-G1) and the *Rsv1* locus have been reported previously in the delimited region on chromosome 13 (Lin et al. 2022; He et al. 2025). To identify haplotype patterns associated with SMV-G1, we conducted local haplotyping within this region, corresponding to the same genomic region studied for SBR on chromosome 13. Phenotyping data for 218 soybean accessions were obtained from a USDA-GRIN evaluation, and accessed from a previous study (He et al. 2025). Local haplotyping was conducted using crosshap v1.4.0 within the region, with parameters epsilon = 0.8 and MGmin = 30.

## Statistical analysis and variant annotation

Welch’s two-sample t-test assuming unequal variances was used to assess differences in disease resistance among populations with unique haplotype categories (Welch 1947). SnpEff was used to annotate variants in MGs of interest (Cingolani et al. 2012). Gene annotations were obtained using JBrowse via SoyBase (Brown et al. 2021).

## Results

### Genome-wide association analysis (GWAS) for soybean rust with SoySNP50K and imputed datasets

**Fig. 1:**
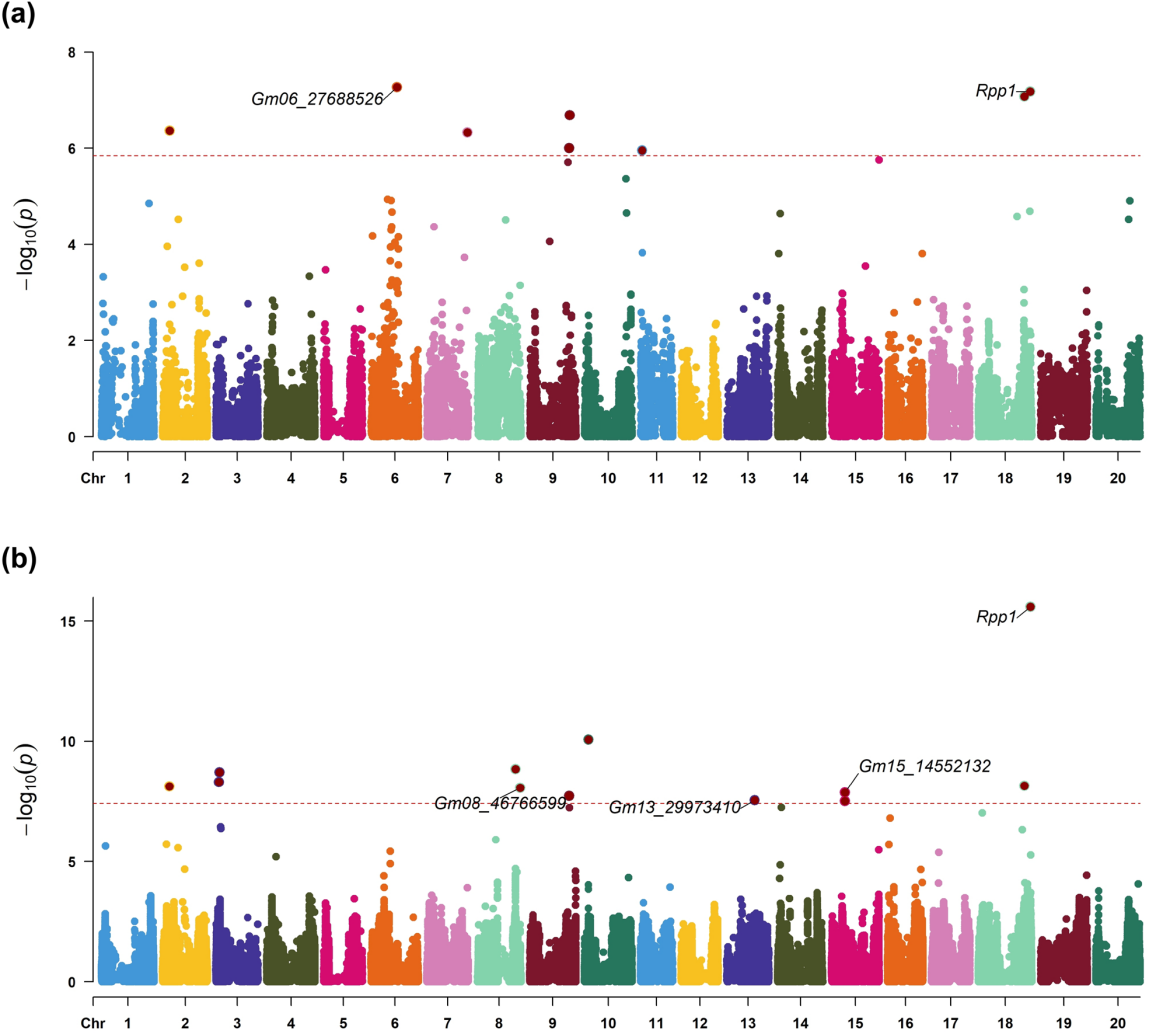
Manhattan plots of genome-wide association analysis (GWAS) for soybean rust resistance obtained using the Fixed and random model Circulation Probability Unification (FarmCPU) model. The plot shows the negative logarithm of association probabilities (− log_10_*p*) values plotted against each single-nucleotide polymorphism position. Significant markers are represented above the red threshold line. Markers of interest are labelled. **a** GWAS results using the SNP-chip array from SoySNP50k **b** GWAS results using an imputed dataset based on SoySNP50K

GWAS using the SoySNP50K data identified eight significant associations across five chromosomes using the FarmCPU model (Table 1, Fig. 1a, Figure S1a). The most significant SNP was mapped to position 27,688,525 on chromosome 6 (Gm06_27688525, *p*-value 5.43 × 10^−8^) on the Wm82.a2.v1 reference genome within the rust resistance QTL reported in the soybean cultivar PI 635999 (Vuong et al. 2016; Lin et al. 2022) (Fig. 1a). Notably, Gm06_27688525 is located 215 kb upstream of a leucine-rich repeat receptor-like kinase 1 gene (*Glyma.06G221400*). The significant SNP at 56,100,116 on chromosome 18 (Gm18_56100116, *p*-value 6.69 × 10^−8^) was previously identified in a GWAS on SBR resistance (Chang et al. 2016). Gm18_56100116 is located within a genomic region that overlaps with multiple reports of the *Rpp1* gene locus (Ray et al. 2009; Yamanaka et al. 2015a) and within the recently identified QTL at the *Rpp1* locus in PI 594756 between 55,863,741 and 56,123,516 bp (Barros et al. 2023). No significant SNPs were identified in the GWAS using the MLM model with SoySNP50K dataset (Figure S2). However, the top SNPs were Gm02_7315227 (*p-*value 5.05 × 10^−6^), Gm20_36935706 (*p-*value 7.23 × 10^−6^), Gm07_43600726 (*p-*value 1.40 × 10^−5^), Gm18_56100116 (*p-*value 1.41 × 10^−5^), and Gm09_42790738 (*p-*value 2.64 × 10^−5^).

**Table 1:** List of markers with significant associations for soybean rust from a genome-wide association study using SoySNP50K genotyping data with the FarmCPU model (*p*-value ≤ 1.42 × 10^−6^)

SNP ID	Chromosome	Position (bp)*	Effect**	*p*-value
Gm06_27688526	Gm06	27,688,526	0.12422	5.43 × 10^−8^
Gm18_56100116	Gm18	56,100,116	−0.19552	6.69 × 10^−8^
Gm18_49673204	Gm18	49,673,204	0.19545	8.39 × 10^−8^
Gm09_43285244	Gm09	43,285,244	−0.13902	2.07 × 10^−7^
Gm02_7315227	Gm02	7,315,227	0.10438	4.35 × 10^−7^
Gm07_43600726	Gm07	43,600,726	−0.13813	4.72 × 10^−7^
Gm09_42790738	Gm09	42,790,738	−0.14232	9.92 × 10^−7^
Gm11_1403993	Gm11	1,403,993	−0.09699	1.12 × 10^−6^

*SNP positions in base pairs with respect to the *G.max* Wm82.a2.v1 reference genome

**magnitude and direction of association between the variant and disease resistance

Association analysis with the imputed dataset revealed 12 loci, including the most significant SNP at 56,472,200 on chromosome 18 (Gm18_56472200, *p*-value 2.49 × 10^−16^) within the *Rpp1* locus (Table S2, Fig. 1b, Figure S1b). There were four significant SNPs in the GWAS with the imputed dataset that overlapped genomic regions identified using the SoySNP50K dataset. These were on chromosome 18 (Gm18_56472200, Gm18_49673204), chromosome 9 (Gm09_42790738), and on chromosome 2 (Gm02_7135106) (Table 1, Table S2).

The significant SNP on chromosome 8 (Gm08_46766599, *p*-value 8.57 × 10^−9^) lies within the rust resistance QTL identified by Harris et al. (2015). The marker on chromosome 15 (Gm15_14552112, *p*-value 3.09 × 10^−8^) is 1.1 Mb away from the resistance loci reported by Walker et al. (2022). A significant marker was identified at position 29,973,410 on chromosome 13 (Gm13_29973410, *p*-value 2.79 × 10^−8^). Notably, the 29–30 Mb region on chromosome 13 has been reported to contain QTLs associated with multiple soybean diseases (Table 2) (Lin et al. 2022). The SNP at 29,973,410 on chromosome 13 likely falls within the SBR locus (qSBR_Gm13) reported previously (97.97–111.88 cM, 11 cM confidence interval) (Harris et al. 2015). Resistance loci for SBR have also been reported at 36,896,763 (Walker et al. 2022) on chromosome 13.

**Table 2:** A list of previously reported quantitative trait loci (QTLs) associated with various soybean diseases in the region 29,880,063–30,239,909 on chromosome 13, based on the Williams 82.a2.v1 reference genome

Disease	QTL name/flanking markers	Marker position (bp)	References
Frogeye leaf spot	ss715614578-ss715615158	28,207,736–31,449,060	(Smith 2021)
Phytophthora root rot	*Gm13_29043806_T_C*	30,125,163–30,154,255	(Scott et al. 2019)
Phytophthora root rot	*QTL-13*	28,842,184–30,776,191	(de Ronne et al. 2020)
Soybean root-knot nematode	BARC-010501–00676, Sct-033	28,826,405–30,078,140	(Xu et al. 2013)
Soybean mosaic virus G1	ss715614803, ss715614847, ss715614951, ss715615024	29,850,000–30,730,000	(He et al.^2025^)

Marker positions are based on the *G.max* Wm82.a2.v1 reference genome. QTL information was compiled from individual studies as cited and from a comprehensive review on soybean disease resistance loci (Lin et al. 2022).

## Linkage disequilibrium analysis and selection of haplotype windows

**Fig. 2:**
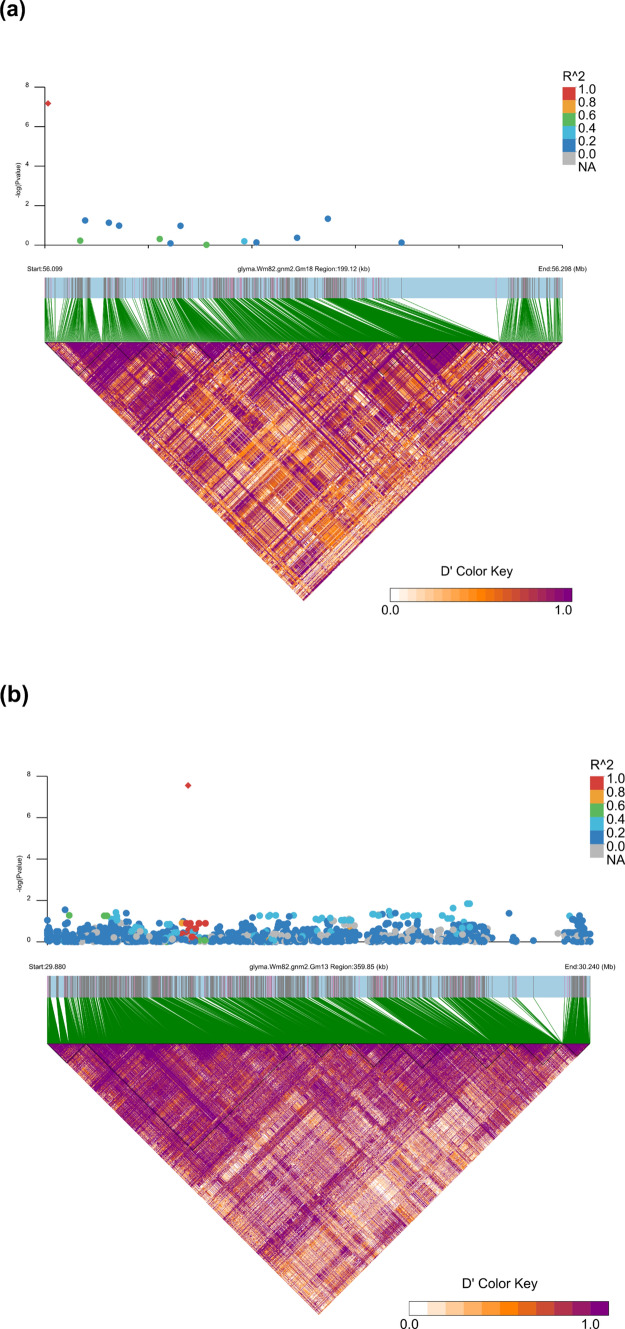
Linkage disequilibrium (LD) patterns (D′ and r^2^) around significant single-nucleotide polymorphisms (SNPs) identified through genome-wide association studies (GWAS) for soybean rust resistance. The top panel displays − log_10_(*p*) values from GWAS, with SNPs coloured by their r^2^ values relative to the lead SNP (orange diamond). The bottom triangle shows D′, with the colour gradient indicating the strength of linkage of all markers with each other in the region. Thin black lines show LD blocks defined using the Solid Spine definition of blocks (Qu et al. 2020) **a** LD in a 199.53 kb around Gm18_56100116 on chromosome 18 **b** LD in a 359.85 kb around Gm13_29973410 on chromosome 13

The significant SNP, Gm18_56100116, shows LD (r^2^ ≥ 0.5) with a cluster of SNPs located within approximately 100 kb of its position (Figure S3, Table S3). Beyond this region, LD decays rapidly, with the majority of SNPs beyond 250 kb showing minimal linkage (r^2^ < 0.4); however, occasional SNPs with r^2^ ≥ 0.6 even at distances of 500–750 kb from Gm18_56100116 were observed. A 199.53-kb region with 939 SNPs (56,098,721–56,298,006) around Gm18_56100116 includes seven main haplotype structures defined by the Solid Spine definition of LD blocks (Qu et al. 2020) (Fig. 2a). This genomic interval also includes the upstream region of the *Rpp1* locus and was delimited for local haplotyping.

On chromosome 13, the lead SNP, Gm13_29973410, is in strong LD (r^2^ ≥ 0.75, Table S4) with several imputed SNPs up to a 107 kb region. A 360-kb region (29,880,063–30,239,909) around Gm13_29973410 with 2,205 SNPs including several haplotype structures and annotated disease resistance genes on Wm82.a2.v1 reference genome was delimited for local haplotyping (Fig. 2b). There are 31 SNPs in this 360-kb region in the SoySNP50K dataset, of which three SNPs at positions 30,067,943, 30,157,326, and 30,163,006 showed weak LD (r^2^ = 0.236, 0.209, 0.230, respectively) with Gm13_29973410.

## Local haplotyping and marker group (MG) composition at the *Rpp1* locus associated with soybean rust on chromosome 18

We initially examined haplotype patterns using a manually selected subset of 23 RB (varying raw RB phenotypic scores) and 43 TAN (raw TAN scores 3.7 −5) by assigning binary scores, excluding individuals with intermediate TAN reactions showing reduced sporulation levels (Table S5). Local haplotyping within the delimited region around Gm18_56100116 with the subset identified two distinct haplotypes: Haplotype A (38 TAN, 1 RB) and Haplotype B (exclusively RB) (Figure S4a). Notably, Haplotype B shows linkage associations with marker groups (MGs; groups of tightly linked SNPs) MG1 (mean r^2^ = 1) and MG5 (mean r^2^ = 1), which carries alternate alleles associated with reduced disease severity (Figure S4a, S4b, S4d).

**Fig. 3:**
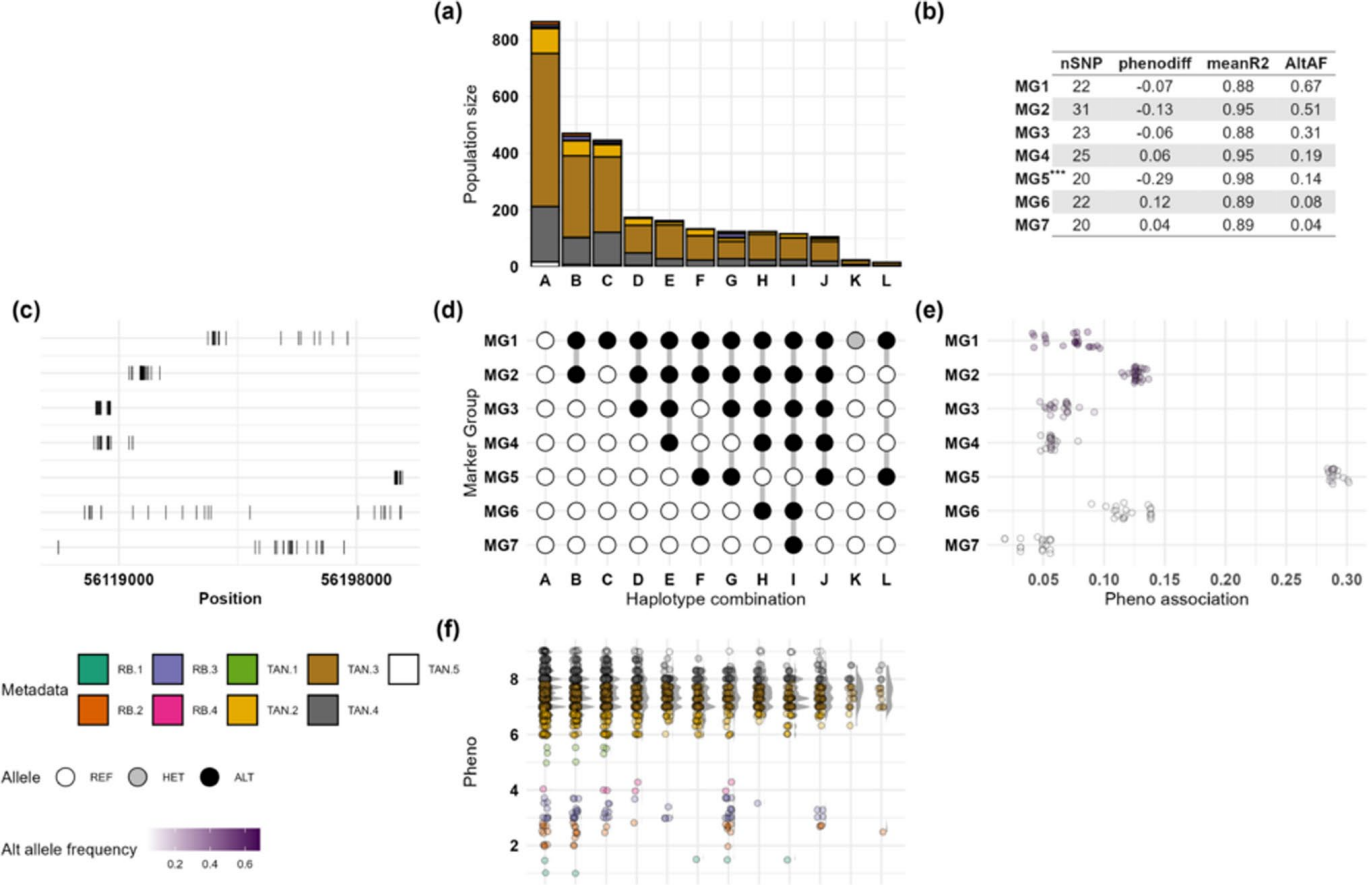
Local haplotyping and phenotypic associations in a 199.53 kb region around the significant single-nucleotide polymorphism (SNP), Gm18_56100116 on chromosome 18 associated with soybean rust. The figure summarises marker groups and their phenotypic effects in the target region. Marker groups (MG1–MG7) represent groups of tightly linked SNPs. Haplotypes A-L show unique allelic combinations of MGs and individuals having them. Main panels include: **a** individual count in each group coloured by metadata **b** summary statistics of each MG, **c** alternate allele frequency per MG, **d** allele distributions across MGs and haplotype groups, **e** phenotypic differences linked to alternate alleles of each MG, **f** phenotype distribution across individual groups. Individuals with alternate alleles for the SNPs in MG5 showed a significant difference (*p*-value = 7.491 × 10^−5^) in phenotype scores compared to those carrying reference alleles. Abbreviations: Alt, alternate allele; AltAf, alternate allele frequency; Het, heterozygous; MISS, missing SNPs; MG, marker group; nSNP, number of SNPs; phenodiff, phenotypic difference for individuals with alternate alleles of MGs vs. reference allele; R^2^, mean linkage correlation between SNPs in an MG; RB.1, metadata grouping individuals with reddish-brown (RB) lesion colour with phenotype scores between 1 and 2; RB.2, phenotype scores between 2 and 3; RB.3, phenotype scores between 3 and 4; RB.4, phenotype scores between 4 and 4.3; Ref, reference allele; SNP, single-nucleotide polymorphism; TAN.1, metadata grouping individuals showing tan (TAN) lesions having phenotype scores between 1 and 2,; TAN.2, phenotype scores between 2 and 3; TAN.3, phenotype scores between 3 and 4; TAN.4, phenotype scores between 4 and 4.9; TAN.5, phenotype scores above 5

To characterise haplotype patterns within the whole population across the full phenotypic range, we conducted local haplotyping within the delimited region around Gm18_56100116, including the entire RB and TAN individuals using the raw phenotype scores. MGs identified in this whole-panel analysis are hereafter referred to by prefixing Gm18 (e.g. Gm18_MG5 refers to MG5 seen in Fig. 3). This analysis identified 12 haplotype combinations (A-L), having allelic combinations for seven MGs (MG1–MG7) (Fig. 3a). Unlike the binary subset analysis that identified two distinct haplotypes, certain haplotypes were observed in both RB and TAN individuals (Fig. 3a, 3d).Approximately 30.76% of the entire population has the reference allele for MG1-MG7. Gm18_MG5 shows a phenotype difference of − 0.29 (mean r^2^ = 0.98, alternate allele frequency = 0.14) (Fig. 3b, Table S6). Comparison of phenotype scores between individuals carrying the alternate versus reference alleles for the SNPs in Gm18_MG5 showed a significant difference (*p*-value = 7.491 × 10^−5^) (Table S7a). Gm18_MG5 appears to have nested linkage patterns with Gm18_MG1 (mean difference in phenotype scores between reference and alternate allele individuals = − 0.07) (Fig. 3d). Individuals having Gm18_MG5 include 32 accessions with RB and 345 with TAN reactions (Table S8). Individuals harbouring known *Rpp1/Rpp1b* alleles such as PI 587905, PI 587880A, and PI 587886 were classed into Haplotype J having alternate alleles for the SNPs in Gm18_MG1 and Gm18_MG5 (Fig. 3a, 3d, Table S8). Haplotype G consists of linked MGs (MG1, MG2, MG3, and MG5) all of which have negative phenotypic differences associated with a reduction in disease severity (Fig. 3a, 3d, 3e). Notably, Gm18_MG6 and Gm18_MG7 showed positive phenotypic differences, indicating alternate alleles are associated with increased disease severity, which were predominantly seen in TAN individuals (Haplotypes H and I). The significant SNP, Gm18_56100116, with a phenotype difference of − 0.448 was not placed in any marker group (Table S6).

To identify variants more specifically influencing resistance within the RB population which itself shows variation in sporulation levels, we conducted an analysis exclusively within RB-phenotyped individuals. Within the RB population, six MGs (MG1-MG6) with tightly linked SNPs (mean r^2^ = 1) and positive phenotypic differences were identified, indicating that alternate alleles are associated with reduced resistance in these individuals (Figure S5, Table S9). Notably, all the six MGs are present in individuals in haplotype group B. These individuals have an average trait mean of 3.07 and include landraces originating from China such PI 437663 (RB = 1.5) and PI 291309C (RB = 2) (Table S10). However, MG7 has a phenotypic difference of − 0.14 (mean r^2^ = 1), indicating that alternate alleles of the SNPs in MG7 are associated with improved resistance. Variant annotation of the SNPs in MG1-MG6 shows the presence of missense, synonymous and downstream gene variants in *Glyma.18G280400* (leucine-rich repeat-containing protein). Downstream gene variants for *Glyma.18G280300* (NB-ARC domain disease resistance protein) and *Glyma.18G280600* (translation initiation factor eIF-2B delta subunit), and upstream gene variants for *Glyma.18G281300* (GNAT acetyletransferases) were also present. Variants for the SNPs in MG7, associated with improved resistance, include *Glyma.18G282000*, an ADP-ribosylation factor (ARF) GTPase-activating protein.

## Local haplotype composition at the chromosome 13 locus associated with soybean rust

Local haplotyping around the delimited region on Gm13_ 29,973,410 within the whole population identified 10 haplotype combinations (A–J) having allelic combinations for five MGs (MG1-MG5) (Figure S6). MG3 is associated with a reduction in disease severity, with negative phenotypic differences of − 0.18 (Figure S6b). Haplotypes G and H have linked MGs including MG3 and are seen in some TAN and RB individuals (Figure S6a, S6d, S6f). Haplotypes I and J appear to consist of linked MGs associated with an increase in disease severity seen in some TAN individuals (MG1, MG4) in the population studied (Figure S6a, S6d).

Local haplotyping within the RB population identified MGs (MG2–MG5) with negative phenotypic differences (Figure S7, Table S11). MG5 with 47 tightly linked SNPs (mean r^2^ = 0.94, alternate allele frequency = 0.04) shows a phenotypic difference of -0.45 (Figure S7b, Figure S7e). Individuals in haplotype group F having linked MGs (MG3, MG5) include accessions with RB phenotype score between 2 and 2.8 (PI 68494, PI 91144, PI 84674, PI 437323). Variants within MG5 are annotated as intronic, upstream or 3′ UTR variants affecting *Glyma.13G185400* (uncharacterised protein), *Glyma.13G185500* (type I inositol 1,4,5-trisphosphate 5-phosphatase CVP2-like)*, Glyma.13G186600* and *Glyma.13G186700* (zinc-induced facilitator-like 1), *Glyma.13G187100* (leucine carboxyl methyl transferase)*,* and *Glyma.13G187200* (COP9 signalosome complex subunit-like protein)*.* Additionally, missense variants of *Glyma.13G187300* which encodes an uncharacterised conserved protein family UPF0565 were identified within the SNPs in MG5. The other MGs also contained missense or synonymous variants for *Glyma.13G185200* (3′(2′),5′-bisphosphate nucleotidase), *Glyma.13G187200*, *Glyma.13G185300* (SUN domain-containing protein 1-like isoform X3), and *Glyma.13G186800* (histone-lysine N-methyltransferase*)*. The significant SNP, Gm13_29973410, was not placed in any marker group, with a phenotype difference of 0.051 (Table S11).

## Marker group transfer from soybean rust resistant (RB) to susceptible (TAN) accessions

**Fig. 4:**
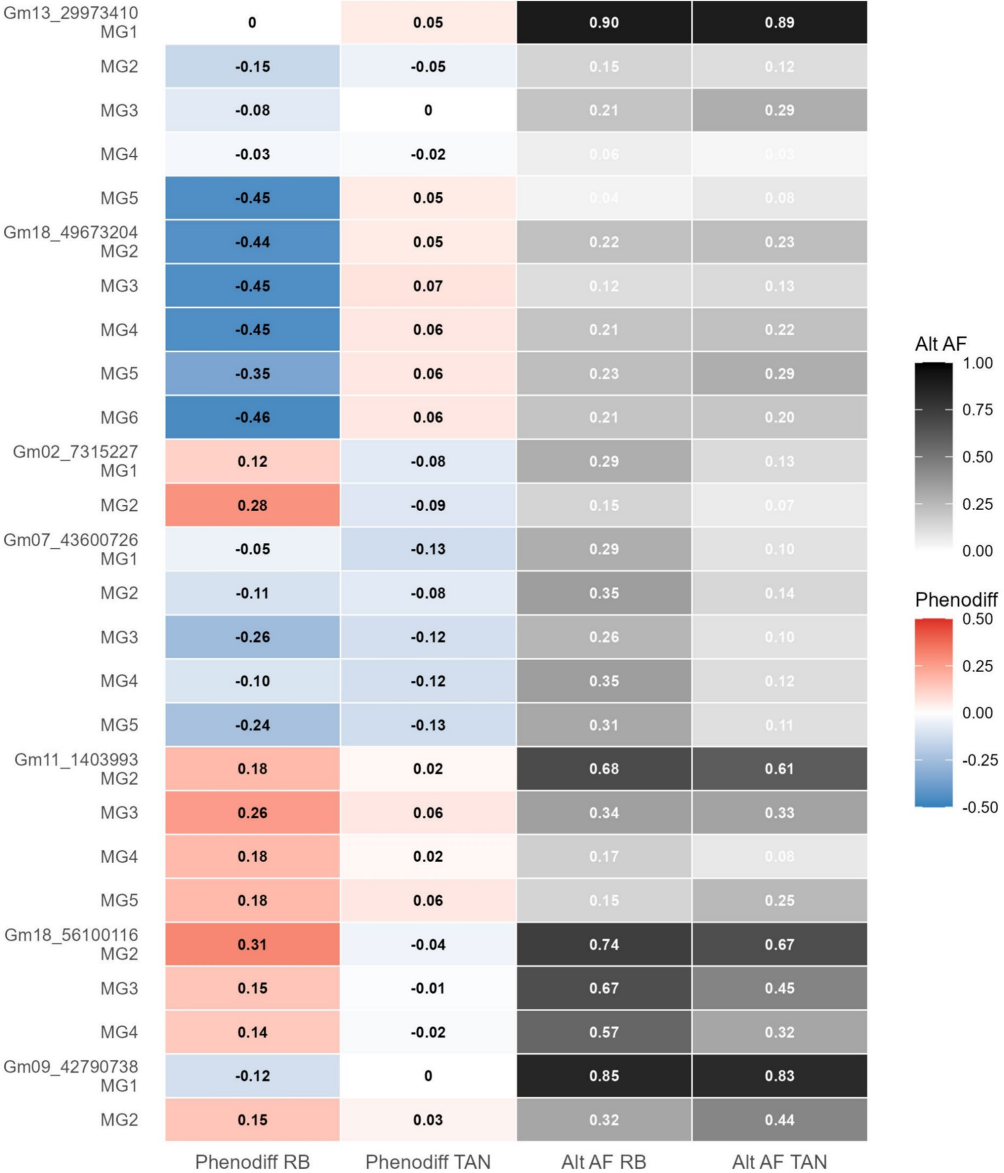
Heatmap of cross-population marker group transfer from soybean accessions with reddish-brown lesions to those with tan lesions (TAN) population at the significant SNPs associated with soybean rust. Each row represents a marker group nested under its respective SNP locus. Phenodiff values are colour coded (blue = associated with reduced disease severity, red = associated with increased disease severity) Abbreviations: Alt AF, alternate allele frequency; MG, marker group with clusters of linked markers identified with local haplotyping; phenodiff, mean phenotype differences between individuals with alternate alleles vs. reference alleles for MGs, negative phenodiff are associated with improved resistance; SNP, single-nucleotide polymorphism

To evaluate whether resistance-associated MGs identified in the RB population confer similar effects in the TAN population, we applied MGs derived from RB haplotyping around all the significant GWAS loci to the corresponding regions in the TAN population. Six loci showed reduced or reversed effects when transferring MGs from RB to TAN backgrounds (Fig. 4). Notably, MGs at Gm07_43600726 which has variants for an autophagy gene, *Glyma.07G261000,* maintained their negative direction in both backgrounds, though with reduced magnitude in TAN. MGs at Gm18_49673204 shifted from high negative phenotype differences in RB to slightly positive values in TAN. MGs at Gm02_7315227 showed opposite effects between populations, with MGs showing positive phenotype differences in RB, but negative values in TAN individuals. At Gm13_29973410, MG2 and MG4 retained their resistance-associated effects in TAN population as well, though with smaller phenotypic differences (− 0.05 and − 0.02, respectively) (Figure S8). However, MG5 which showed negative phenotypic differences in RB individuals showed a reversal effect with positive phenotypic differences (0.05) in TAN. Alternate allele frequencies were generally lower in the TAN population compared to RB across most loci, except at Gm18_56100116 where frequencies remained high in both populations, with contrasting phenotypic effects.

## Local haplotyping in the targeted region on chromosome 13 for soybean mosaic virus (SMV-G1)

**Fig. 5:**
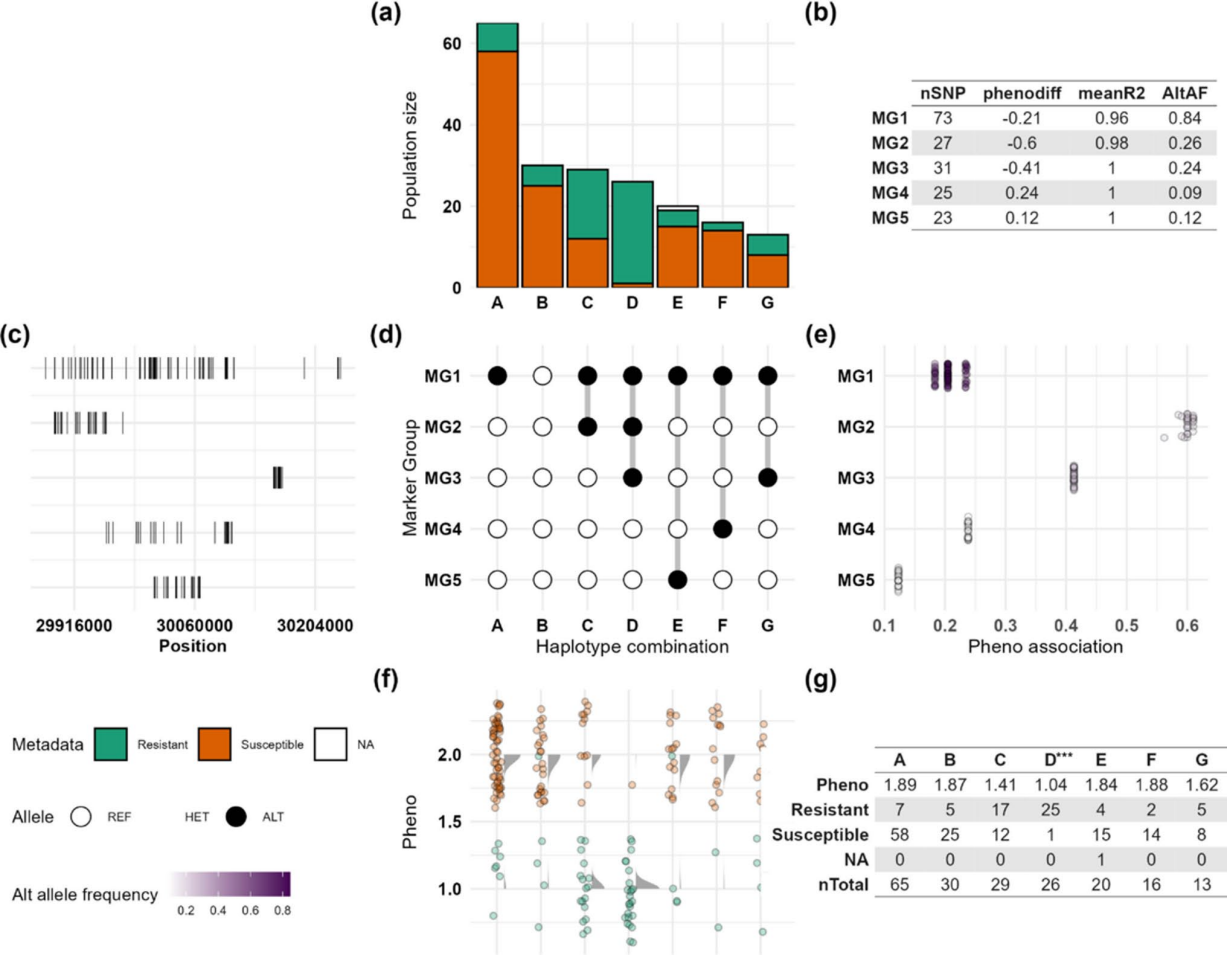
Local haplotyping and phenotype associations in a 360-kb region around the marker Gm13_29973410 on chromosome 13, in the soybean mosaic virus resistant and susceptible populations. The figure summarises marker groups (MGs) and their phenotypic effects in the region. MG1–MG5 represent groups of tightly linked SNPs; groups A–G indicate unique haplotype combinations, defined by distinct allelic states across all MGs and shared by individuals in that group (Marsh et al. 2023). Main panels include: **a** individual count in each group coloured by metadata, **b** summary statistics of each MG, **c** SNP positions per MG, **d** allele distributions across MGs and haplotype groups, **e** phenotype differences linked to alternate alleles of each MG, **f** phenotype distribution across individual groups, and **g** summary table. Comparison of phenotype scores between haplotypes showed a significant difference for individuals with haplotype D (*p*-value = 2.2 × 10^−16^) Abbreviations: Alt, alternate allele; AltAf, alternate allele frequency; Het, heterozygous; MG, marker group; nSNP, number of SNPs; phenodiff, phenotype difference for individuals with alternate alleles of MGs vs. reference allele, negative phenodiff are associated with improved resistance; R^2^, mean linkage correlation between SNPs in an MG; Ref, reference allele; SNP, single-nucleotide polymorphism

Local haplotyping in the focused region around Gm13_29973410 for soybean mosaic virus shows five MGs (MG1-MG5) (Fig. 5, Table S12). Of these, MG1, MG2, and MG3 show negative phenotype differences (− 0.21, − 0.6, − 0.41, respectively) (Fig. 5b, 5d). Haplotype D with 25 resistant and one susceptible individual (PI 506883) having the alternate alleles at SNPs in MG1, MG2, and MG3 appears as a distinct SMV-resistant haplotype combination (Fig. 5a, 5d, 5f, Table S13). Comparison of phenotype scores between haplotypes groups showed a significant difference (*p*-value = 2.2 × 10^−16^, Table S7b) for individuals carrying haplotype D, seen in resistant individuals. PI 96983, which carries the single dominant *Rsv1* gene against SMV (Hajimorad and Hill 2001), is also grouped into haplotype D (Table S13). The variants in haplotype D include several genes, including missense variants for *Glyma.13G186400*, and intronic variants for *Glyma.13G184900*, a candidate gene for *Rsv1* (Table S14a).

## Discussion

### Allelic diversity and haplotype complexity at the* Rpp1* locus associated with soybean rust (SBR) within a diverse germplasm

Previous studies dissecting allelic diversity at the *Rpp1* locus have largely focused on known *Rpp1/Rpp1b* sources, susceptible checks, or derived breeding lines within limited sample sets (Kim et al. 2012; Barros et al. 2023; Aoyagi et al. 2024). For instance, Aoyagi et al. (2024) examined a 100-sample panel enriched in resistant (62) *Rpp1/Rpp1b* sources, characterising allelic patterns at seven GWAS-derived SNPs. They identified the GTC allelic pattern in a subset of RB accessions including in PI 587880A. The GTC pattern served as a diagnostic marker and validated to distinguish resistant from susceptible plants in a biparental mapping population, showing its breeding application for the introgression of the *Rpp1-b* resistance from PI 587880A. Even within such a restricted panel, the TTT pattern was shared in some RB and TAN accessions, highlighting the allelic complexity at this locus. LD between the three SNPs forming the GTC pattern was low (r^2^ = 0.26–0.38), and none of these SNPs were within candidate causal genes the authors identified. In a global germplasm of 1,511 soybean accessions, the GTC pattern was observed only in 16 accessions (Aoyagi et al. 2024), potentially carrying the *Rpp1-b* allele. Similarly, Barros et al. (2023) identified SNPs that can be successfully applied to select lines bearing the *Rpp*-allele from PI 594756 for breeding purposes.

Building on these previous studies, we examined linkage structures around the *Rpp1* locus in an unselected, phenotypically diverse population, to identify durable sources of genetic variation. In a manually selected subset of 65 accessions, assigned binary phenotypic scores (RB = 1, TAN = 2), removing intermediate TAN reactions from the analysis, we identified two distinct haplotypes present in either RB or TAN individuals. While this demonstrates the potential of LD-based haplotyping for diagnostic purposes, it does not capture the true biological reality of allelic diversity at this locus. It also ignores the possibility of identifying genetic variation such as partial resistance present in TAN individuals. Whole-population haplotyping using raw phenotypic scores for all RB and TAN individuals revealed the complexity and fine-scale allelic diversity present at this locus in the population studied, identifying 12 haplotype combinations. The marker group, Gm18_MG5 (r^2^ = 0.98), significantly associated with a reduction in disease severity, was seen in TAN (342) and RB (32) individuals.

Population-specific haplotyping within the RB individuals revealed distinct haplotype structures associated with reduced rust resistance on chromosome 18. The variant annotation shows missense variants for *Glyma.18G280400,* a candidate R2 gene at the *Rpp1* locus. The other variant, *Glyma.18G280300,* is a candidate R1 gene at the *Rpp1* locus (Pedley et al. 2019; Barros et al. 2023) and is also associated with resistance to *Phytophthora sojae* (Hodge et al. 2025). The co-occurrence of these gene variants with variants in a translation initiation factor (*Glyma.18G280600*) suggests that these haplotypes (MG2, MG3) may be associated with reduced resistance through a coordinated mechanism involving pathogen recognition and translational control. Notably, several members of the translation initiation factor family, such as eIF4E and eIF4G, are well-established susceptibility factors for plant viruses and have been shown to mediate resistance or susceptibility depending on their allelic states (Sanfaçon 2015; Shopan et al. 2017). In contrast, gene variants in MG7 associated with improved resistance, *Glyma.18G282000*, are a candidate gene encoding an ARF1 protein. Small GTPases, such as ARF1, have been shown to be involved in non-host resistance and R-gene-mediated resistance in plants (Coemans et al. 2008). Further experimental validation is required to clarify the specific roles of these gene variants on SBR resistance, particularly the candidate gene, *Glyma.18G282000.*

Overall, our analysis at the *Rpp1* locus provides the following insights: i) Distinct haplotypes exclusive to RB or TAN can be identified using LD-based local haplotyping in a panel with known resistant or susceptibility checks; however, this obscures the true allelic diversity at this locus in an unselected population. ii) Resistance-associated MGs (Gm18_MG5) appear in both RB and TAN individuals, suggesting potential sources of durable resistance. iii) MGs associated with increased disease severity (Gm18_MG6, Gm18_MG7) occur in some, but not all TAN individuals, indicating genetic heterogeneity in susceptibility. iv) Within RB, MGs (MG1-MG6) are associated with increased disease severity, including variants for *Glyma.18G280400, Glyma.18G280300,* and *Glyma.18G280600,* indicating the potential for resistance improvement by selecting or stacking favourable haplotype combinations.

## Resistance-associated soybean rust variation patterns within a multi-pathogen defence region on chromosome 13

We examined a 360 kb region (29.8–30.2 Mb) around a rust resistance significant marker identified that likely encompasses the qSBR_Gm13 mapped by Harris et al. (2015) previously, with the whole population and within RB individuals only. In the RB-only haplotyping, MG5, a marker group associated with reduced rust severity, was identified within this region that contains tightly linked variants for candidate resistance genes beyond traditional R genes. Among these genes, the zinc-induced facilitator-like (ZIFL) genes (*Glyma.13G186500*/*600*/*700*) belong to the Major Facilitator Superfamily and are commonly involved in auxin transport regulation and drought response (Pao et al. 1998; Remy et al. 2013). In *Brassica napus*, two candidate ZIFL genes (*BnaCO3g0610D* and *BnaCO3g06020D*) were highly upregulated in resistant lines during *Sclerotinia sclerotiorum* infection (Qasim et al. 2020). Transgenic studies have shown that basal defence and systemic acquired resistance response are affected in plants with altered inositol phosphatase (Hung et al. 2014), suggesting a potential role for the Type 1 inositol 5-phosphatase candidate gene *Glyma.13G185500* in SBR resistance. Soybean inositol 5-phosphatase is also induced under salt stress with improved plant tolerance when overexpressed (Jia et al. 2020). Recent phosphoproteomic studies in soybean have shown that overexpressing both phosphomimic and phosphodead variants of the candidate SUN1 protein, *Glyma.13G185300,* significantly reduced susceptibility to soybean cyst nematode infection (Hawk et al. 2024). Another candidate gene, *Glyma.13G187200,* encodes a COP9 signalosome complex subunit-like protein associated with plant defence responses, particularly jasmonic acid synthesis (Hind et al. 2011). These findings suggest that the candidate non-R genes may contribute to SBR resistance through diverse molecular mechanisms at the chromosome 13 locus. Future functional validation focusing on these non-R candidate genes is required, and it may provide valuable insights for developing durable resistance to SBR.

## Distinct resistance haplotypes associated with soybean mosaic virus around the *Rsv1* locus on chromosome 13

Distinct SMV-resistant haplotype D with marker group combinations (MG1, MG2, MG3) at the *Rsv1* locus was detected, when haplotyping was performed across the entire population. *Rsv1* locus is a well-studied complex resistance gene locus containing multiple NBS-LRR genes and confers extreme resistance to SMV strains, G1–G6 (Hayes et al. 2004; Wu et al. 2019). This locus shows significant allelic and gene diversity among cultivars, with different combinations of these genes conferring varying phenotype resistance reactions (Zhang et al. 2012; Usovsky et al. 2022). Within such a complex locus, the candidate genes can evolve, leading to positive selection within a resistant population, giving rise to functional resistance genes that are clearly divergent from susceptible individuals (Ma et al. 2018; Wu et al. 2019). In fungal or bacterial defence response, plants typically activate pattern-triggered immunity (PTI) upon the recognition of pathogen-specific molecular patterns such as bacterial flagellin or fungal chitin. In contrast, viral defence mainly involves R-gene-mediated resistance (such as NBS-LRR genes) and RNA-silencing pathways (Moon and Park 2016). Therefore, R-gene-mediated resistance such as conferred by *Rsv1* is highly specific and effective, compared to the more diffuse and gradual quantitative resistance variation observed in SBR. However, there is increasing recognition of the role of PTI and other related defence signalling components in antiviral responses (Kørner et al. 2013; Moon and Park 2016; Huang et al. 2023). For instance, pathogen recognition receptors such as malectin-like receptor kinases have been shown to enhance SMV resistance at the *Rsv4* locus on chromosome 2 (Che et al. 2023). Similarly, double-stranded RNA, in addition to inhibiting viral replication, can trigger PTI-like responses through receptor-like kinases (Huang et al. 2023). Therefore, PTI- and NBS-LRR-mediated immunity may act synergistically rather than independently, as shown through recent studies (Calil and Fontes 2017; Ngou et al. 2022).

In our study, the SMV-resistant haplotype combination D (comprising MG1, MG2, MG3) around *Rsv1* includes gene variants that have potential functional roles in a defence response: primary R-gene candidates (*Glyma.13G184900*), PTI-associated helper components such as S-domain receptor kinases (*Glyma.13G188800*) (Tang et al. 2017), regulatory and signalling components such as MYB transcription factors (e.g. *Glyma.13G188700*) (Yu et al. 2023), proteolytic components such serine proteases (*Glyma.13G187000*) (van der Hoorn and Klemenčič 2021), and cellular homeostasis components, including zinc-induced facilitators (multiple genes: *Glyma.13G186700/600/500/400*) (Meena et al. 2021). This multi-layered composition of the resistant haplotypes aligns with recent studies on SMV resistance, which is increasingly considered to be from the action of a network of genes more than just NBS-LRR genes. For instance, recent studies have shown that other than primary R-genes, SMV resistance requires helper signalling components such as EDS1, PAD4, as well as transcription factors (e.g. WRKY3, ERF) and hormone signalling through jasmonic and salicylic acid pathways (Zhang et al. 2012; Ross et al. 2021; Li et al. 2023). Since PI 96983, an *Rsv1* carrier, also contains haplotype D, it suggests that these tightly linked gene variants may contribute to resistance along with R genes in this accession (Gore et al. 2002). Together, these findings suggest that SMV resistance may be governed by a multi-layered defence strategy, supporting previous studies (Gore et al. 2002; Zhang et al. 2012), and consistent with the gene composition observed in our SMV-resistant haplotype. However, future functional validation of these resistance-specific variants is required to confirm their precise roles in conferring SMV resistance.

Although this genomic region on chromosome 13 harbours resistance loci for both SBR and SMV, our findings suggest that underlying haplotype structures (with respect to the linked SNPs in the MGs) are distinct and pathogen specific. However, comparing the gene variants within the resistance-associated haplotypes identified for SBR and SMV showed that zinc-induced facilitators (*Glyma.13G186500*/*600*/*700/800*), *Glyma.13G185500*, and *Glyma.13G185300* were common to haplotypes associated with both the diseases (Table S14). For *Glyma.13G185300,* the variation patterns differed between the two diseases. For SBR, variants (between 29,921,986 and 29,930,656) were mainly upstream, with low allele frequencies, including a synonymous variant (SNP position, 29,927,307). SMV haplotypes showed higher allele frequencies (e.g. alternate allele frequency = 0.80 at 29,925,329, T > C) for the same gene variant (between 29,922,529 and 29,928,308) with mainly upstream gene variants and an intronic SNP (A > C) at 29,928,308. Similarly, *Glyma.13G185500* variants showed higher alternate allele frequencies in SMV-associated haplotypes, with variants mainly in upstream regions and 3′/5′ UTRs for both diseases. These shared gene variants suggest their role in broader defence response in soybean; however, future functional validation is needed to confirm their roles.

## Context-dependent local haplotype effects for soybean rust resistance and implications for breeding

Our results from cross-population haplotype transfer analysis show that when resistance-associated MGs from the RB individuals were transferred into the TAN population, there was a reduction or even reversal of its phenotypic effect around most significant SNPs. These patterns suggest that haplotype effects for SBR resistance may be context dependent, with the same haplotype showing different phenotype outcomes based on interactions between variants and the surrounding genetic background. Spontaneous or induced mutations can result in variable phenotypic effects depending on genetic backgrounds (Goldstein and Ehrenreich 2021). Such background effects can lead to incomplete penetrance, where mutations show effects only in certain individuals, or variable expressivity, where effects differ across individuals (Griffiths 2005; Goldstein and Ehrenreich 2021). These may contribute to different degrees of expression of a quantitative trait in a population based on the mutations they possess (Goldstein and Ehrenreich 2021). Mutation effects may also be influenced by epistatic interactions with alleles present at other loci (Alcázar et al. 2009). For instance, in barley *(Hordeum vulgare* L.), resistance conferred by *Mla6* gene against powdery mildew fungus, *Blumeria graminis f. sp. hordei,* requires the *SGT1* cofactor for its effect. Individuals carrying the same *Mla6* haplotype may show different phenotypic effect if the *SGT1* is non-functional (Chapman et al. 2021). In our study, phenotype variation seen in soybean rust resistance despite identical haplotypes may therefore result from epistasis, copy number variation, environmental effects, or quantitative genetic effects (Alcázar et al. 2009; McHale et al. 2012; Bonde et al. 2013; Doust et al. 2014; Mullis et al. 2018).

On chromosome 07, MGs identified around Gm07_43600726 retained resistance effects in both RB and TAN individuals, though with reduced magnitude in TAN. These MGs include variants in an autophagy-related gene, *Glyma.07G211600*. Autophagy is an evolutionarily conserved process in higher plants, defects in which can result in immunity-related programmed cell death (Yoshimoto et al. 2009). However, in Arabidopsis, a loss of function for autophagy gene, *ATG2*, improves resistance to biotrophic fungal pathogens through elevated salicylic acid (SA) and reactive oxygen intermediates, particularly hydrogen peroxide (H_2_O_2_) (Yoshimoto et al. 2009; Lenz et al. 2011; Wang et al. 2011). In soybean, there are two *ATG2* homologues, *Glyma.02G133400* and *Glyma.07G211600,* referred to as *GmATG2* (Hashimi et al. 2021). Silencing *GmATG2* mediated by *Bean pod mottle virus* led to accelerated yellowish leaf senescence under dark conditions as reported by Hashimi et al. (2021). However, silencing also led to an accumulation of *GmATG8*, poly-ubiquitinated proteins, bound salicylic acid (SA), and H_2_O_2_ (Hashimi et al. 2021). Notably, Hashimi et al. (2021) observed that *GmATG2* silenced soybean showed improved resistance to the biotrophic pathogen, *Pseudomonas Syringae pv. Glycinea* (*Psg*) with significant expression of Pathogen-Related (PR) genes. *P. pachyrhizi* is an obligate biotrophic fungus that is sensitive to SA-mediated defence responses (Glazebrook 2005; Campe et al. 2014). Biotic agents such as acibenzolar-S-methyl can trigger systemic acquired resistance through SA accumulation, and synthesis of PR proteins, reducing SBR infection severity (Cruz et al. 2013; Oliveira et al. 2016; de Paula et al. 2021). Therefore, it is likely that rust resistance associated with *Glyma.07G211600-*linked candidate haplotypes in our study may be from autophagy disruption and accumulation of SA/PR levels. However, future functional validation of the variants in these MGs is required to determine whether partial loss of function, reduced expression, or silencing *Glyma.07G211600* influences soybean rust resistance.

Pyramiding or combining multiple genes or QTLs using marker-assisted selection can enhance biotic and abiotic stress responses in most crops (Zheng et al. 2017; Yang et al. 2018; Ramalingam et al. 2020; Jin et al. 2023; Cruppe et al. 2023). However, introgressed QTLs do not always give the expected phenotype improvements in the resulting lines, as such introgressions can result in multiple fitness effects (Yadav et al. 2019; Dagilis and Matute 2023). This may be due to interactions of the QTLs with the genetic background, epistasis with neighbouring alleles, environmental factors, or linkage between the introgressed loci (Xu and Crouch 2008; Bovill et al. 2010; Wang et al. 2014; Sandhu et al. 2018; Yadav et al. 2019). These interactions can result in positive, negative, or neutral outcomes depending on the genetic context and selection pressures (Sandhu et al. 2018; Yadav et al. 2019).

Similar context dependence has also been reported for haplotypes. In maize, Tong et al. (2022) introgressed various *ZmCCT* haplotypes that influence both stalk-rot resistance and photoperiod sensitivity, into seven elite maize lines, and developed NILs differing only at *ZmCCT*. NILs having the *ZmCCT* haplotype H5 consistently showed improved yield, drought tolerance and stalk-rot resistance across different genetic backgrounds (Tong et al. 2022). However, the percentage reduction in disease index by H5 varied based on the genetic background (Tong et al. 2022). This shows that introgression of a favourable haplotype (H5) can improve performance, but the improvement should be tested in the target genetic background. The potential of haplotype-based crop improvement has also been shown in a recent study, where introgression of a superior haplotype from the *OsIRO2-H3* gene led to yield advantage of 25–27.3% over recurrent parents in rice (Singh et al. 2025). Similarly, in rice, introgressing blast resistance genes such as *Pi54* improved disease resistance against *Magnaporthe oryzae* and yield for elite lines compared to the recurrent parent (Dileep Kumar et al. 2025). However, multi-environment field testing for other lines carrying *Pi54* showed variable performance which the authors attributed to epistasis and the complexities of diverse pathogen populations in different environments (Dileep Kumar et al. 2025).

Gene expression is thus frequently regulated by multiple regulatory alleles acting together; however, most GWAS analyses do not account for the possibility that phenotype outcomes at a given locus may result from the interaction of such multiple alleles (Corradin et al. 2014; Qian et al. 2016). Our findings suggest that divergent rust resistance among individuals with identical haplotypes may be due to background-dependent effects, which may not be adequately captured by single SNP markers. This further highlights the limitations of single-variant markers and the need for integrated approaches to optimise haplotype-based soybean rust resistance breeding efforts.

While haplotyping has revealed detailed insights into allelic diversity around important loci associated with SBR, it is important to acknowledge that in our study, haplotype analysis was conducted using imputed genotype data. This allowed for the identification of wider variants, including rare alleles, not captured by the original SoySNP50K array. However, imputation-based associations rely heavily on the quality and size of the reference panel, and the accuracy of the imputation process (Deng et al. 2021). Therefore, the haplotype variant findings around trait-associated loci for SBR and SMV should be considered preliminary and hypothesis generating. Further experimental validation in independent populations using targeted sequencing and functional assays is required to confirm the involvement of the identified candidate variants in soybean rust resistance. Analysis of expression patterns of candidate genes such as *Glyma.18G282000, Glyma.13G187200,* and *Glyma.07G261000* at 0, 24, 48, and 72h post-SBR infection using qPCR would help to verify whether they are pathogen induced, to support the functional association identified in our study. Additionally, QTLs for days to maturity have also been reported on chromosome 13 (29,022,554–31,262,263) (Copley et al. 2018) and at 30,271,391 (qDTM-24) (Perfil Ev et al. 2024). Future biparental mapping populations where maturity is controlled as a covariate are necessary to identify true resistance effects in the region studied here. With the availability of updated soybean genome assemblies such as Wm82.a6 (Espina et al. 2024), physical coordinates and structural context of genomic regions studied here can be further refined in future work. Moreover, the seedling plant resistance for SBR considered here may not match adult plant resistance in the field. The impact of genotype-by-environment interactions on rust resistance has not been accounted for in the study as well.

Future studies can build on these findings by examining insertions, deletions, and other structural variants around trait-associated regions that may reveal additional genetic variation influencing soybean rust resistance (Marsh et al. 2022a). Disease resistance predictions integrating genomic data with other multi-omics data (e.g. transcriptomics, metabolomics) using machine learning may provide deeper insights into SBR resistance mechanisms (Mohamedikbal et al. 2025). While our cross-population haplotype transfer analysis uses phenotypic mean differences, alternate allele frequencies, and SNP effect annotations as an observational and informative step to identify beneficial haplotypes, it represents an initial exploratory investigation. Given the availability of more comprehensive multi-environment phenotypic data for soybean disease resistance, future studies could employ more rigorous statistical modelling frameworks such as those developed previously to quantitatively assess haplotype effects and their stability across diverse populations and environments (Millet et al. 2016; Mayer et al. 2020). Additionally, computational simulations to understand the impact of stacking optimal resistance haplotypes may contribute to haplotype-based breeding strategies (Tong et al. 2024; Villiers et al. 2024).

## Conclusion

In this study, we examined allelic diversity to understand the genetic basis of soybean rust and soybean mosaic virus resistance using association analyses and local haplotyping, identifying resistance-associated haplotypes. We have thoroughly assessed linkage structures and haplotype variation patterns at the *Rpp1* locus on chromosome 18 associated with soybean rust, identifying candidate gene variants associated with reduced resistance (e.g. *Glyma.18G280400, Glyma.18G280300,* and *Glyma.18G281300)* and others associated with improved resistance (*Glyma.18G282000)*. On chromosome 13, we examined a shared multi-disease locus identifying distinct haplotypes linked to rust and soybean mosaic virus resistance, with candidate genes including NBS-LRRs, signalling proteins and cellular homeostasis components. For soybean mosaic virus strain G1, we identified a distinct resistance-associated haplotype with gene variants including *Glyma.13G184900, Glyma.13G188800,* and *Glyma.13G187000.* Cross-population haplotype transfer analysis for rust-associated regions showed the variable phenotypic effects of identical haplotypes across genetic backgrounds, with *Glyma.07G261000* maintaining its resistance-associated effects. These findings identify candidate variants for targeted gene editing and highlight the potential of haplotype-informed breeding strategies for durable disease resistance in soybean to meet growing food demands. However, future functional validation of candidate variants and the durability of resistance-associated haplotypes under diverse environmental conditions and pathogen pressure is required.

## Consent to participate

Not applicable.

## Consent for publication

Not applicable.

## Ethical approval

Not applicable.

## Supplementary Information

Below is the link to the electronic supplementary material.

Supplementary file1 (DOCX 1679 KB)

Supplementary file2 (XLSX 652 KB)

## Data Availability

Soybean rust phenotype data along with lesion type for 2,815 accessions were downloaded from USDA-GRIN database, (https://npgsweb.ars-grin.gov/gringlobal/method?id=492634, accessed 06 September 2024). Genotype data for 2,815 accessions were obtained from the SoySNP50K iSelect Beadchip dataset aligned to Wm82.a2.v1 from SoyBase https://data.soybase.org/Glycine/max/diversity/Wm82.gnm2.div.Song_Hyten_2015/glyma.Wm82.gnm2.div.Song_Hyten_2015.vcf.gz (Song et al. 2013, 2015). Reference panel aligned to Wm82.a2.v1 for genotype imputation was obtained from https://data.soybase.org/Glycine/max/diversity/Wm82.gnm2.div.Valliyodan_Brown_2021/ (Valliyodan et al. 2021). The Wm82.a2.v1 reference genome is available at SoyBase. Phenotype data for soybean mosaic virus strain G1 is available at USDA-GRIN https://npgsweb.ars-grin.gov/gringlobal/descriptordetail?id=51159. The code and scripts used to generate results are available at https://github.com/shameelaikbal/soybean_rust_smv_resistance_genomics
