# Supplementary material for: Dissection of local haplotype diversity at soybean rust loci reveals resistance-associated and context-dependent variation patterns in diverse germplasm
Source: Theor Appl Genet. 2026 Mar 25;139(4):107. doi: 10.1007/s00122-026-05209-6 (PMC13018045; doi:10.1007/s00122-026-05209-6)
Supplement: Supplementary file 1 — Supplementary file1 (DOCX 1679 KB) [file 122_2026_5209_MOESM1_ESM.docx]

Supplemental Figures for

**Dissection of local haplotype diversity at soybean rust loci reveals resistance-associated and context-dependent variation in diverse germplasm**

Shameela Mohamedikbal^1,2^, Hawlader A. Al-Mamun^1,2,3^, Jacob I. Marsh^4^, Shriprabha R. Upadhyaya^1,2^, Jacqueline Batley^2^, David Edwards^1,2^

**Author Affiliations**

^1^Centre for Applied Bioinformatics, University of Western Australia, Perth, Western Australia, 6009, Australia

^2^School of Biological Sciences, University of Western Australia, Perth, Western Australia, 6009, Australia

^3^InterGrain Pty Ltd, Perth, Western Australia, 6163, Australia

^4^Department of Biology, University of North Carolina, Chapel Hill, NC 27599, USA

**Corresponding author**

David Edwards, Centre for Applied Bioinformatics and School of Biological Sciences, University of Western Australia, Perth, Western Australia, 6009, Australia.

E-mail: [Dave.Edwards@uwa.edu.au](mailto:Dave.Edwards@uwa.edu.au)

**(a)**


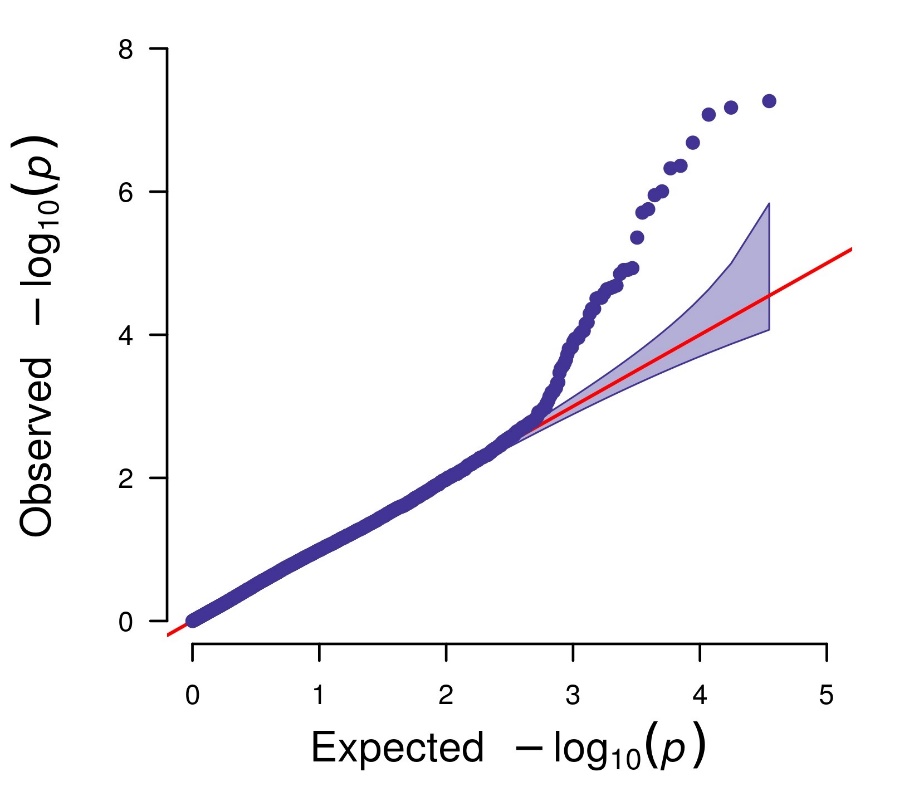


**(b)**


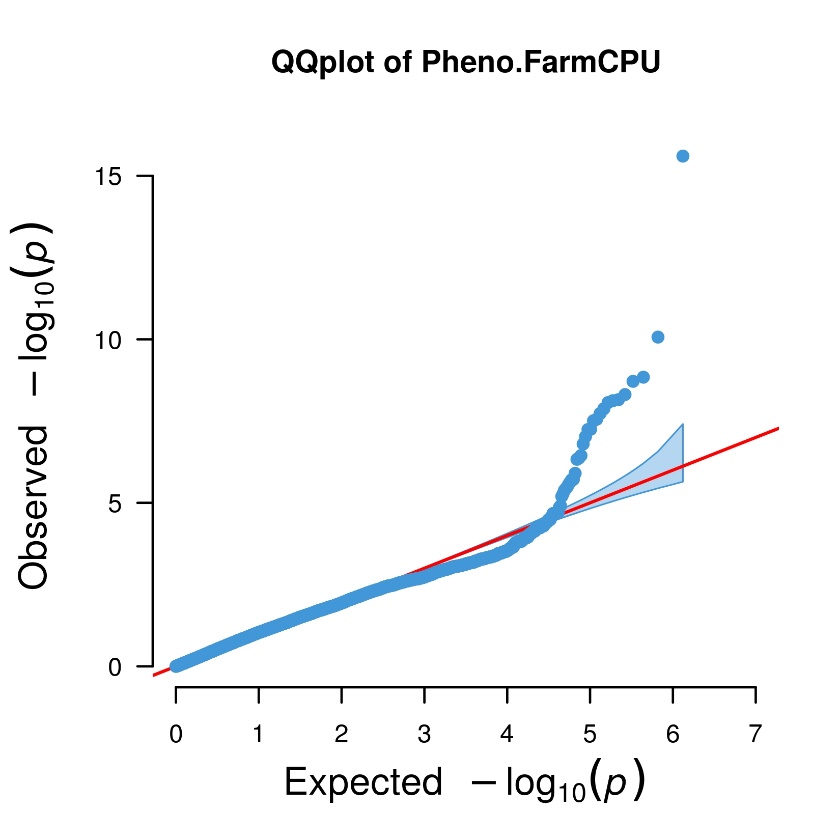


**Figure S1:** Quantile-Quantile (QQ) plot showing the observed p-values plotted against the expected values under the null hypothesis. **a)** QQ plot for the association analysis for soybean rust resistance conducted using SoySNP50K dataset **b)** QQ plot for the association analysis for soybean rust resistance conducted using an imputed dataset


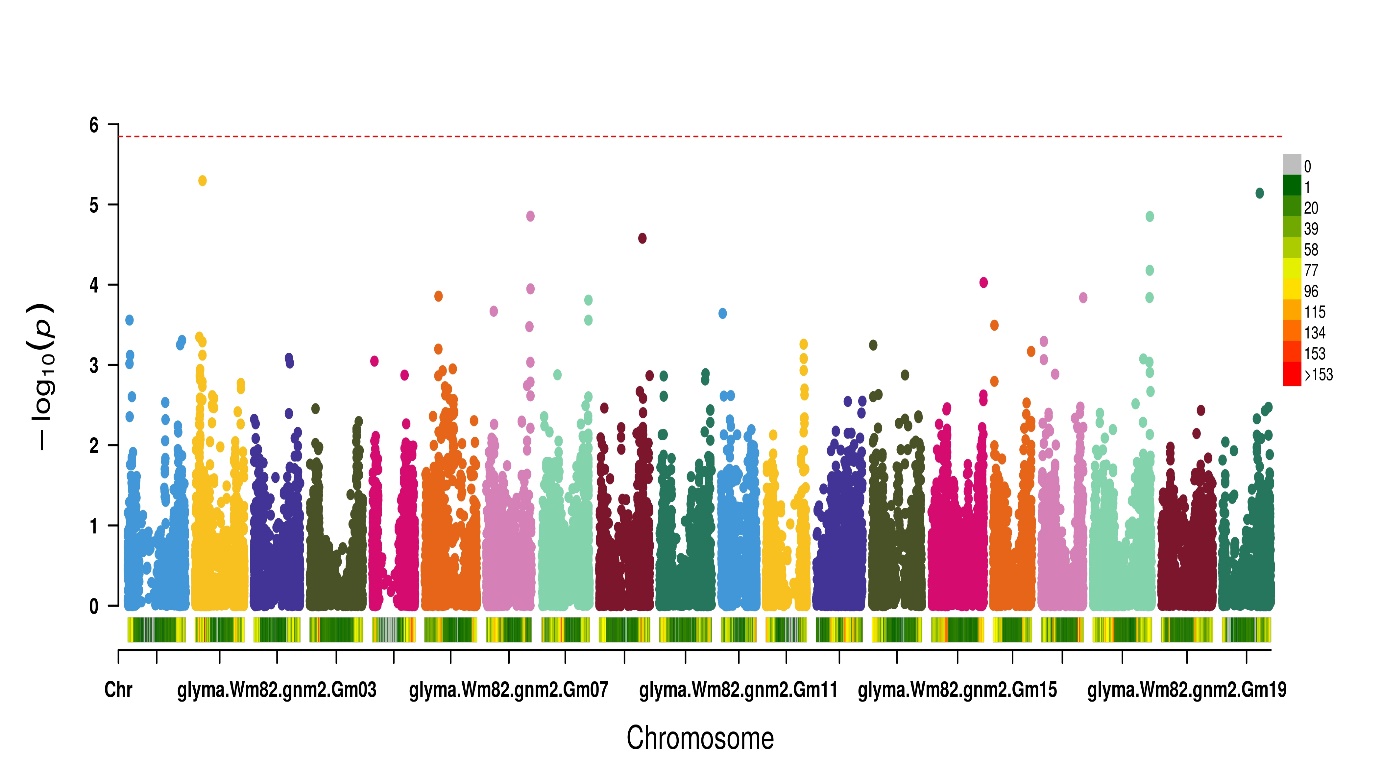


**Figure S2:** Manhattan plots of genome-wide association analysis (GWAS) for soybean rust resistance obtained using a Mixed Linear Model using SoySNP50K. The plot shows the negative logarithm of association probabilities (-log_10_*p*) values plotted against each single nucleotide polymorphism position


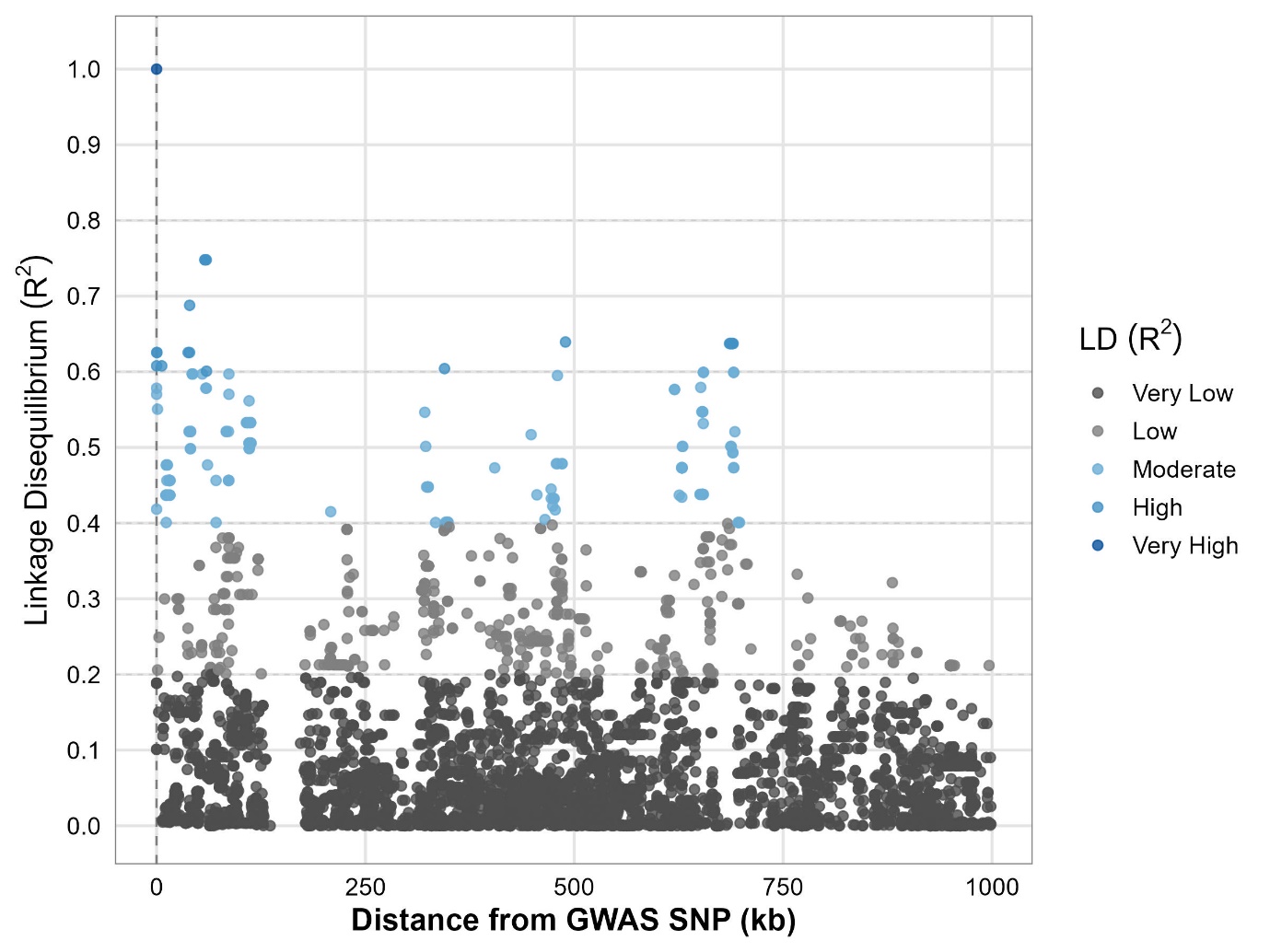


**Figure S3**: Linkage disequilibrium (LD) correlation (R^2^) of significant marker 56,100,116 on chromosome 18 with all the other markers in a 1 Mb region. R^2^ is categorised as Very High (R^2^≥0.8), High (0.6 ≤ R² < 0.8), Moderate (0.4 ≤ R² < 0.6), Low (0.2 ≤ R² < 0.4), Very Low (R² < 0.2). The x-axis shows distance in kb from the lead marker.


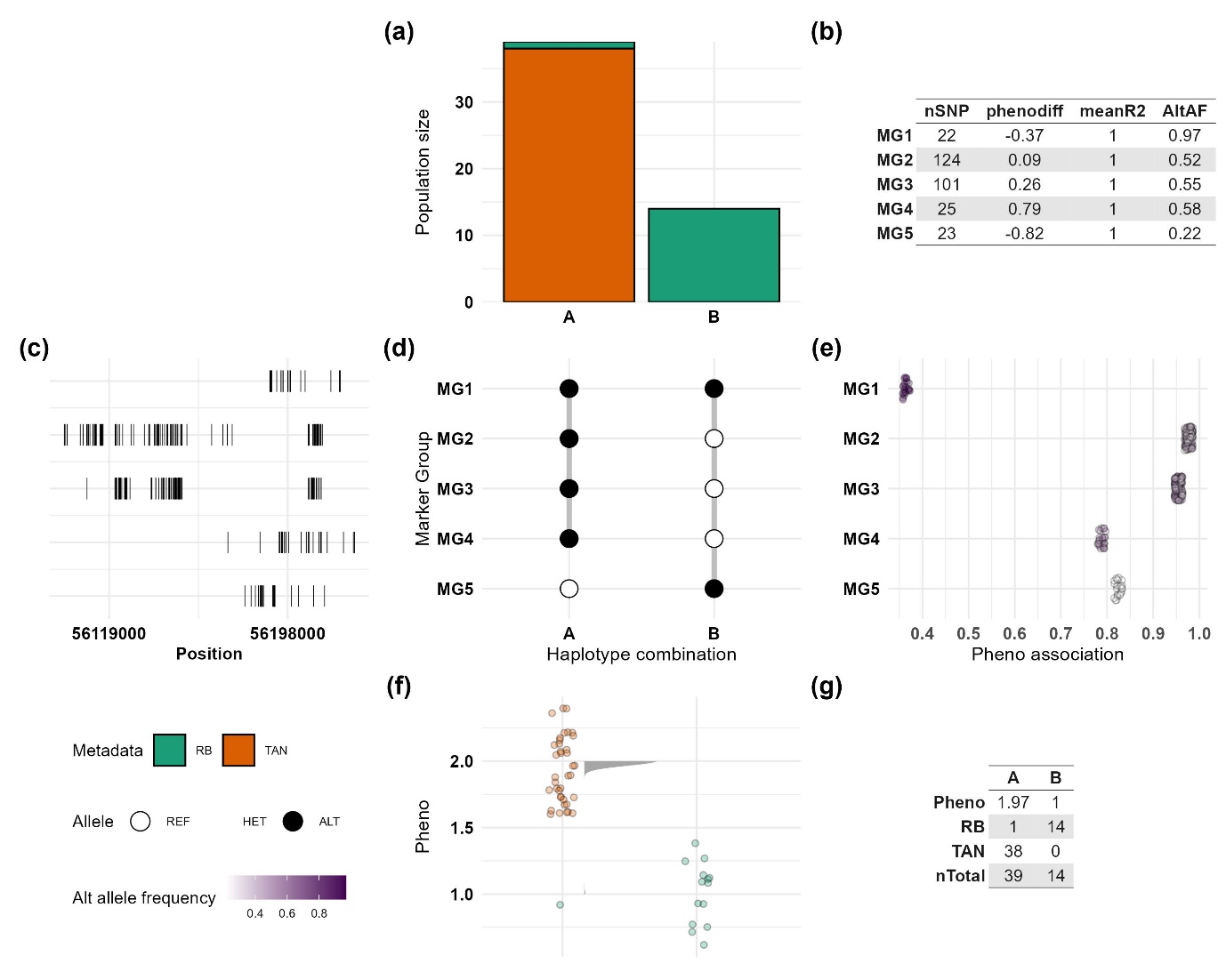


**Figure S4:** Local haplotyping and phenotypic associations in a 199.53 kb region around the significant SNP Gm18_56100116 within a subset (67 accessions) of soybean rust resistant (RB) and susceptible (TAN) population. The figure summarises marker groups (MG1-MG5) and shows two distinct haplotypes (A-B) within this subset. Main panels include: **(a)** individual count in each group coloured by the metadata of lesion colour (RB/TAN), **(b)** summary statistics of each MG, **(c)** alternate allele frequency per MG, **(d)** allele distributions across MGs and haplotype groups, **(e)** phenotypic differences linked to alternate alleles of each MG, **(f)** phenotype distribution across individual groups **Abbreviations:** Alt, alternate allele; AltAf, alternate allele frequency; Het, heterozygous; MISS, missing SNPs; MG, marker group; nSNP, number of SNPs; phenodiff, phenotypic difference for individuals with alternate alleles of MGs vs. reference allele; R^2^, mean linkage correlation between SNPs in an MG


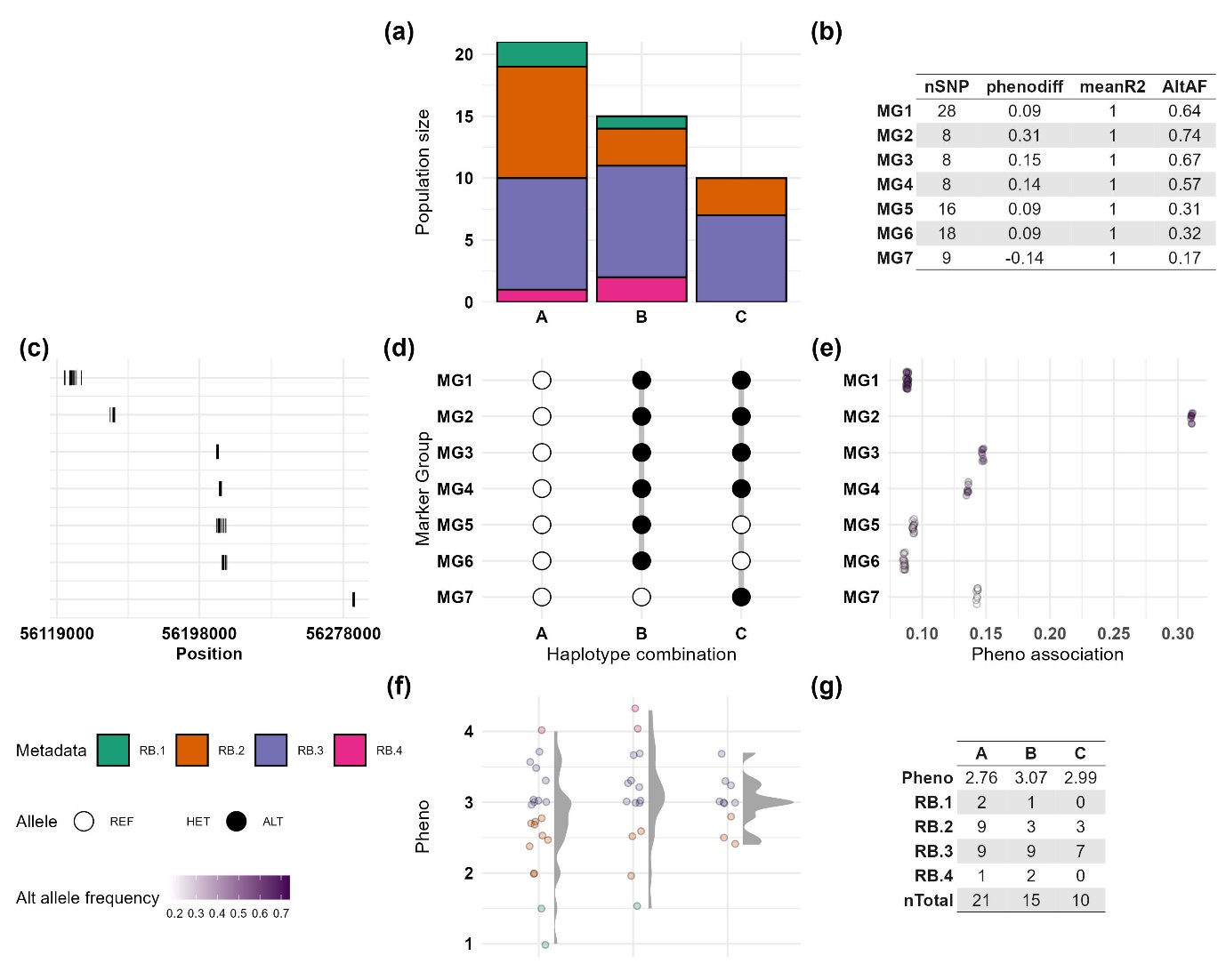


**Figure S5:** Local haplotyping and phenotype associations in a 199.53 kb region around the significant marker, Gm18_56100116, on chromosome 18 in the soybean rust resistant (RB) population using an imputed dataset based on the SoySNP50K dataset. The figure summarises Marker Groups (MGs) and their phenotypic effects in the region. MG1 – MG7 represent groups of tightly linked SNPs; groups A-C indicate unique haplotype combinations, defined by distinct allelic states across all MGs and shared by individuals in that group [(Marsh et al. 2023)](https://sciwheel.com/work/citation?ids=16242253&pre=&suf=&sa=0&dbf=0). Main panels include: **(a)** individual count per haplotype group coloured by metadata, **(b)** summary statistics of each MG, **(c)** SNP positions per MG, **(d)** allele distributions across MGs and haplotype groups, **(e)** phenotype differences linked to alternate alleles of each MG **(f)** phenotype distribution across groups, and **(g)** summary table. **Abbreviations:** Alt, alternate allele; AltAf, alternate allele frequency; Het, heterozygous; MG, marker group; nSNP, number of SNPs; phenodiff, phenotype difference for individuals with alternate alleles of MGs vs. reference allele, with negative phenodiff associated with reduced resistance; R^2^, mean linkage correlation between SNPs in an MG; RB.1, metadata category grouping individuals having resistant phenotype score between 1-2; RB.2, resistant phenotype score between 2-3; RB.3, resistant phenotype score between 3-4; RB.4, resistant phenotype score between 4-5; Ref, reference allele; SNP, single nucleotide polymorphism


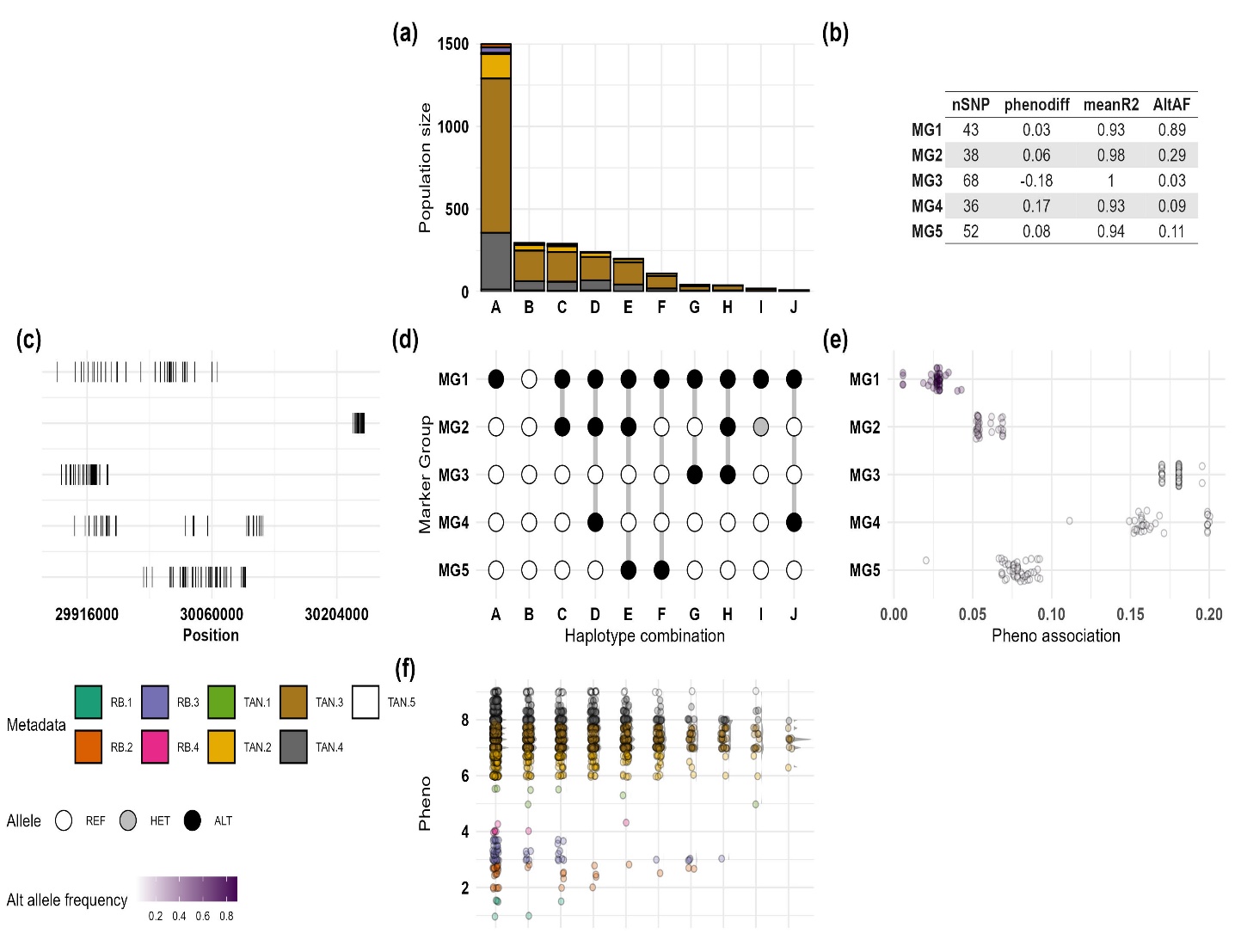


**Figure S6:** Local haplotyping and phenotypic associations in a 360 kb region around the significant SNP Gm13_29973410 associated with soybean rust. The figure summarises marker groups and their phenotypic effects in the target region. Marker groups (MG1 – MG5) represent groups of tightly linked SNPs, while groups A-J show combinations of individuals having them. Main panels include: **(a)** individual count in each group coloured by metadata, **(b)** summary statistics of each MG, **(c)** SNP positions per MG, **(d)** allele distributions across MGs and individual groups, **(e)** phenotypic differences linked to alternate alleles of each MG, **(f)** phenotype distribution across individual groups, and **(g)** summary table. **Abbreviations:** Alt, alternate allele; AltAf, alternate allele frequency; Het, heterozygous; MG, marker group; nSNP, number of SNPs; phenodiff, phenotypic difference for individuals with alternate alleles of MGs vs. reference allele; R^2^, mean linkage correlation between SNPs in an MG; RB.1, metadata grouping individuals with reddish-brown (RB) lesion with phenotype score between 1-2; RB.2, phenotype score between 2-3; RB.3, phenotype score between 3-4; RB.4, phenotype score between 4-5; Ref, reference allele; SNP, single nucleotide polymorphism, TAN.1, metadata grouping individuals with TAN phenotype scores between 1-2, ; TAN.2, TAN phenotype scores between 2-3; TAN.3, TAN phenotype scores between 3-4; .4, TAN phenotype scores between 4-4.9; TAN.5, TAN phenotype scores above 5.


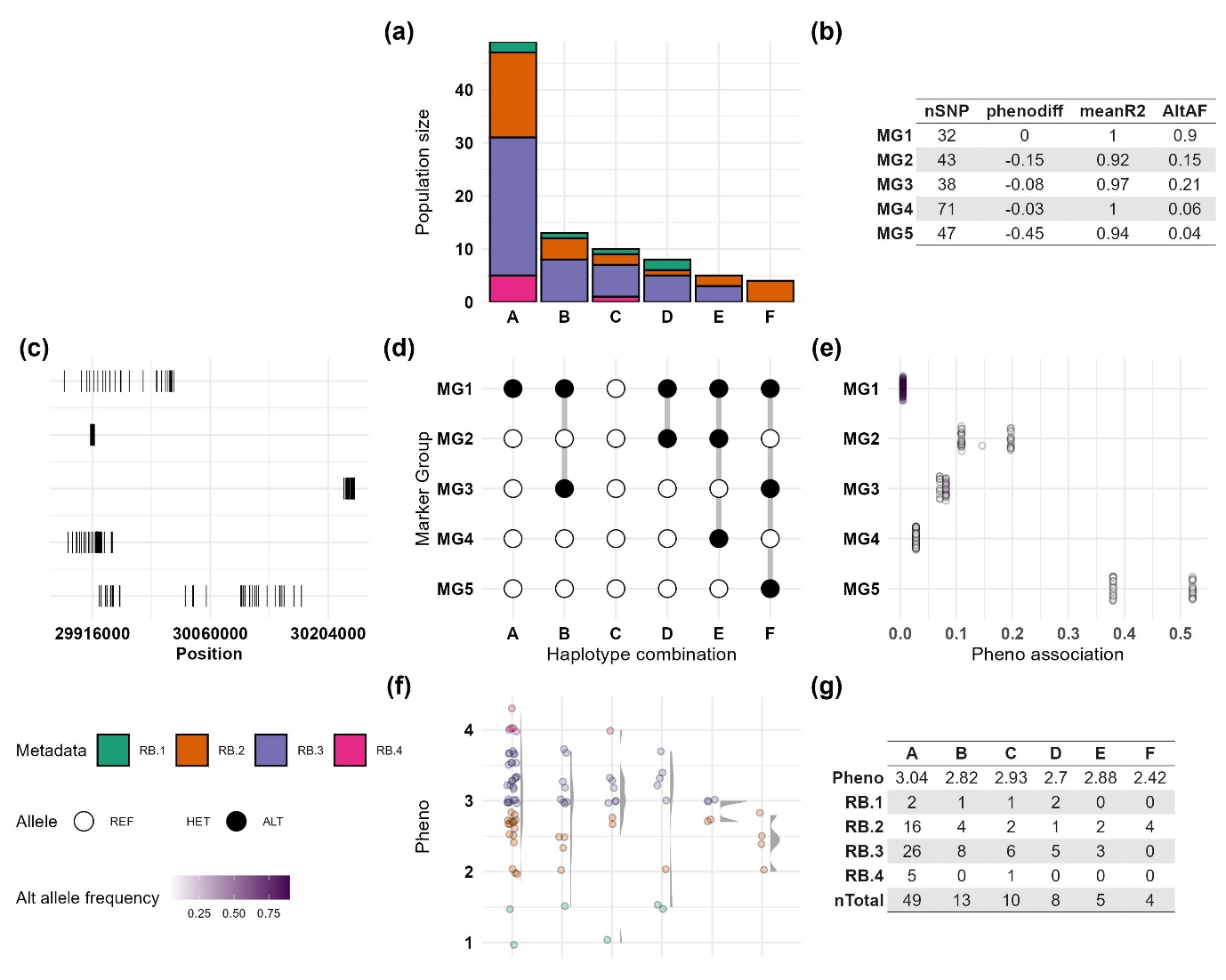


**Figure S7:** Local haplotyping and phenotypic associations in a 360 kb region around the significant SNP Gm13_29973410 within the soybean rust resistant population using an imputed dataset ((DR^2^ ≥ 0.8). The figure summarises marker groups and their phenotypic effects in the target region. Marker groups (MG1 – MG5) represent groups of tightly linked SNPs, while groups A-G show combinations of individuals having them. Main panels include: **(a)** individual count in each group coloured by metadata, **(b)** summary statistics of each MG, **(c)** SNP positions per MG, **(d)** allele distributions across MGs and individual groups, **(e)** phenotypic differences linked to alternate alleles of each MG, **(f)** phenotype distribution across individual groups, and **(g)** summary table. **Abbreviations:** Alt, alternate allele; AltAf, alternate allele frequency; Het, heterozygous; MG, marker group; nSNP, number of SNPs; phenodiff, phenotypic difference for individuals with alternate alleles of MGs vs. reference allele; R^2^, mean linkage correlation between SNPs in an MG; RB.1, metadata grouping individuals with reddish-brown (RB) lesion with phenotype score between 1-2; RB.2, phenotype score between 2-3; RB.3, phenotype score between 3-4; RB.4, phenotype score between 4-5; Ref, reference allele; SNP, single nucleotide polymorphism.


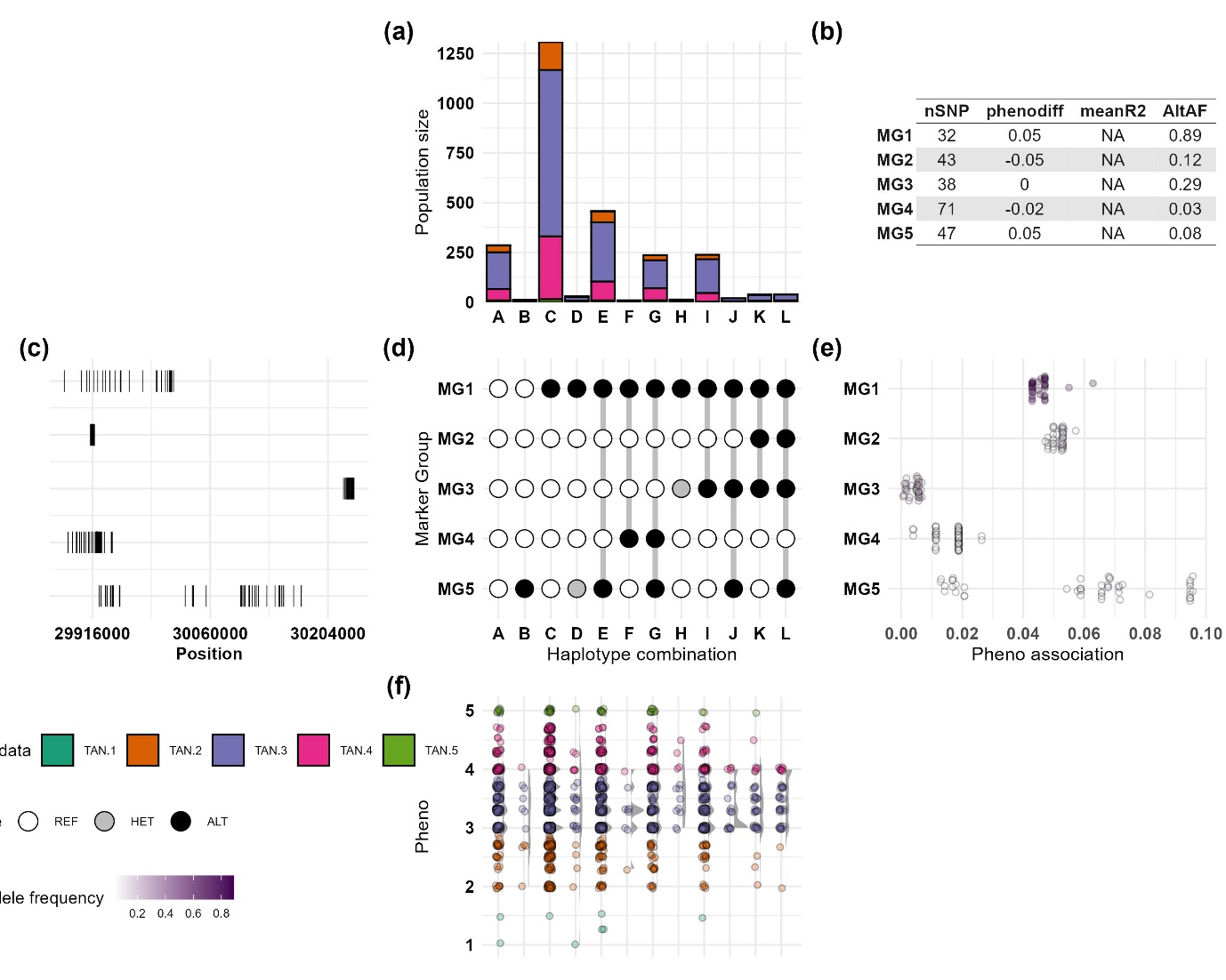
**Figure S8:** Haplotype combinations and phenotypic associations in a 360 kb region around the significant marker Gm13_29973410 within the soybean rust susceptible (TAN) population using the marker groups derived from a resistant population. The figure summarises marker groups and their phenotypic effects in the target region. Marker groups (MG1 – MG5) represent groups of tightly linked SNPs, while groups A-O show haplotype combinations and individuals having them. Main panels include: **(a)** individual count in each group coloured by metadata, **(b)** summary statistics of each MG, **(c)** SNP positions per MG, **(d)** allele distributions across MGs and haplotype groups, **(e)** phenotypic differences linked to alternate alleles of each MG, and **(f)** phenotype distribution across individual groups. **Abbreviations:** Alt, alternate allele; AltAf, alternate allele frequency; Het, heterozygous; MG, marker group; nSNP, number of SNPs; phenodiff, phenotypic difference for individuals with alternate alleles of MGs vs. reference allele; R^2^, mean linkage correlation between SNPs in an MG; Ref, reference allele; SNP, single nucleotide polymorphism, TAN.1, metadata grouping individuals with TAN phenotype scores between 1-2, ; TAN.2, TAN phenotype scores between 2-3; TAN.3, TAN phenotype scores between 3-4; .4, TAN phenotype scores between 4-4.9; TAN.5, TAN phenotype scores above 5.
